# Non-Coding RNAs in Colorectal Cancer: Their Functions and Mechanisms

**DOI:** 10.3389/fonc.2022.783079

**Published:** 2022-02-02

**Authors:** Zimo Jia, Jiaqi An, Ziyuan Liu, Fan Zhang

**Affiliations:** ^1^ Department of Biochemistry and Molecular Biology, Hebei Medical University, Shijiazhuang, China; ^2^ School of Medicine, Shihezi University, Shihezi, China; ^3^ The Key Laboratory of Neural and Vascular Biology, Ministry of Education, Shijiazhuang, China

**Keywords:** ncRNAs, colorectal cancer, function, mechanism, tumorigenesis

## Abstract

Colorectal cancer (CRC) is a common malignancy with high mortality. However, the molecular mechanisms underlying CRC remain unclear. Controversies over the exact functions of non-coding RNAs (ncRNAs) in the progression of CRC have been prevailing for multiple years. Recently, accumulating evidence has demonstrated the regulatory roles of ncRNAs in various human cancers, including CRC. The intracellular signaling pathways by which ncRNAs act on tumor cells have been explored, and in CRC, various studies have identified numerous dysregulated ncRNAs that serve as oncogenes or tumor suppressors in the process of tumorigenesis through diverse mechanisms. In this review, we have summarized the functions and mechanisms of ncRNAs (mainly lncRNAs, miRNAs, and circRNAs) in the tumorigenesis of CRC. We also discuss the potential applications of ncRNAs as diagnostic and prognostic tools, as well as therapeutic targets in CRC. This review details strategies that trigger the recognition of CRC-related ncRNAs, as well as the methodologies and challenges of studying these molecules, and the forthcoming clinical applications of these findings.

## Introduction

Colorectal cancer (CRC), with a high incidence and mortality rate, is the third most prevalent malignant tumor and the second leading cause of cancer-related death worldwide (1). Globally, there are two main distinct pathways of precursor lesions: the conventional adenomatous carcinoma pathway (also known as the chromosomal instability sequence) leading to 70%-90% of CRC, and the serrated neoplasia pathway leading to 10%-20% of CRC (2). These pathways represent a diverse multiplicity of genetic and epigenetic events in a fairly consistent sequence (3). The chromosomal instability phenotype typically develops after a genomic event triggered by an APC mutation, followed by RAS activation or function loss of TP53. In contrast, the serrated neoplasia pathway is associated with RAS and RAF mutations and epigenetic instability characterized by a CpG island methylation phenotype, leading to microsatellite stable and unstable cancers (2). Due to the lack of distinctively incipient symptoms and the limitations of early detection, most CRC patients are diagnosed at advanced stages. Metastatic disease accounts for the vast majority of cancer-associated deaths. And the liver is the most frequent site of distant metastasis from CRC, with over 50% of CRC deaths being attributed to metastasis (4). Despite advances in the diagnosis and treatment of CRC, such as some practical chemotherapeutic drugs and immunotherapy, the genetic background and underlying molecular mechanisms mediating this disease are still unclear (5). Although the increased risk of toxicity and cost, new therapeutic agents have exactly improved survival in advanced disease settings. However, the long-term prognosis for metastatic disease remains poor due to late diagnosis and treatment failure (6). To improve CRC therapy, novel potent drugs require identification, and therapeutic strategies need to be appraised and developed.

The result of the human genome project shows that protein-coding genes represent less than 2% of the total human genome, whereas, more than 90% of the human genome is composed of non-coding RNAs (ncRNAs), which are actively transcribed from the human genome but cannot encode proteins (7, 8). ncRNA families are habitually divided into two broad categories. One is housekeeping ncRNA, including highly abundant ribosomal RNAs (rRNAs) and transfer RNAs (tRNAs). The other is regulatory ncRNAs, including long ncRNAs (lncRNAs), microRNAs (miRNAs), circular RNAs (circRNAs), PIWI-interacting RNA, tRNA-derived small RNA (tRFs), small nucleolar RNA (snoRNAs), siRNAs, and so on (9). Among the most studied classes of ncRNAs are lncRNAs, miRNAs, and circRNAs.

As one of several types of ncRNAs, lncRNAs are larger transcripts with more than 200 nucleotides (nt) in length, and a considerable portion of lncRNAs species are transcribed by polymerase II. Similarly, most have 5’-end m^7^G caps and 3’-end poly(A) tails, and they are presumed to be transcribed and synthesized in the same way as mRNAs, but cannot translate into protein (10). There are two types of functional elements in lncRNAs, one is the interactor element that directly interacts physical with other molecules, and proteins, and the other is the structural element, which leads to the formation of secondary and/or tertiary 3D RNA structures and regulates their functional interactions (11). It is the ability to interact with RNA and proteins through rigorous base pairing or chemical interactions in secondary structures that enables lncRNAs to function in various ways. Many lncRNAs have been identified to play a role in gene regulation, for instance, by affecting transcription factor targeting or epigenetic modification. In addition, interactions with mRNAs might cause the alteration of their transcriptional speed and stability. As such, the interaction between lncRNAs and proteins may affect protein activity, stability, or localization (12, 13).

**Figure 1:**
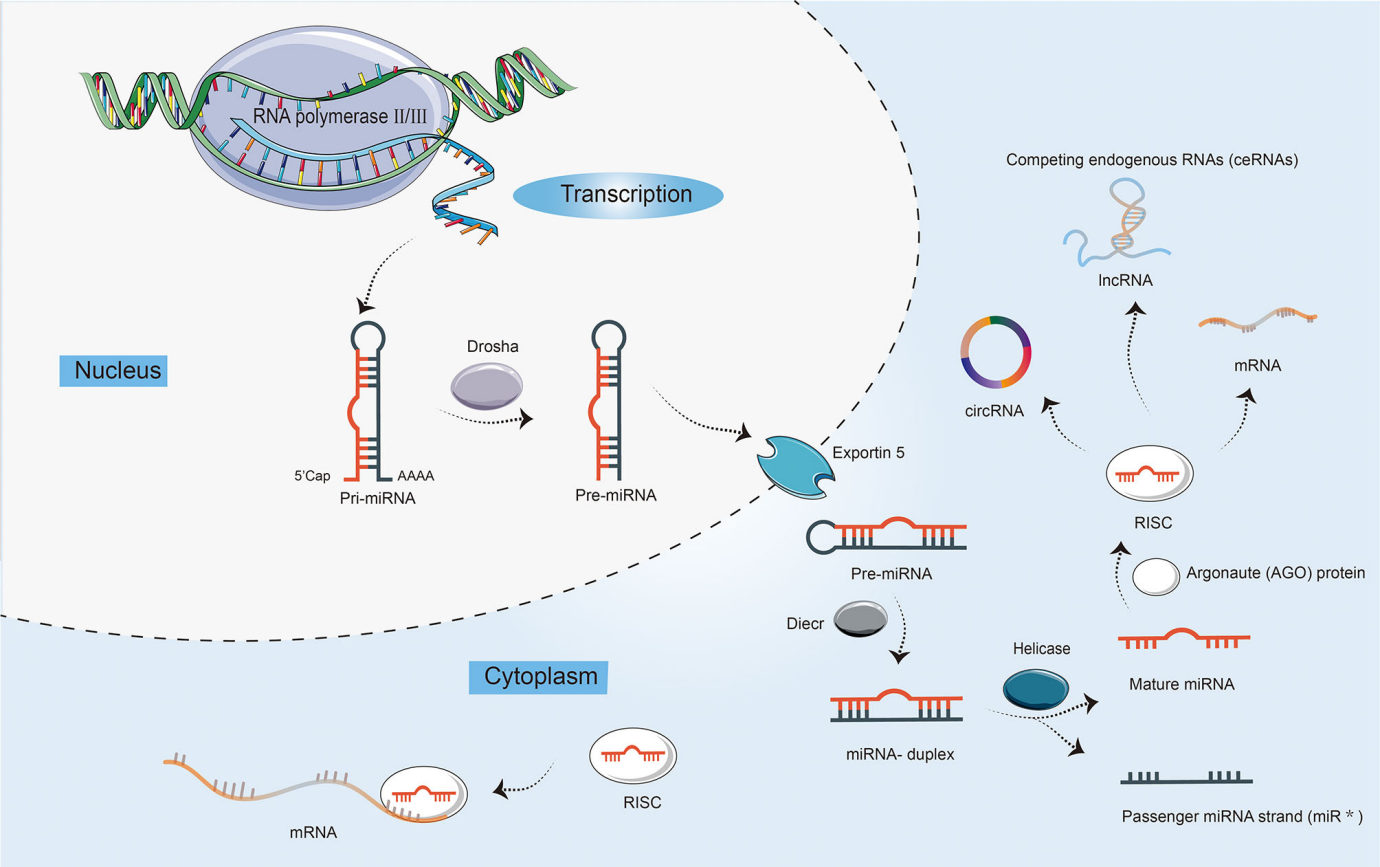
The biogenesis and characteristics of microRNAs (miRNAs). First, the transcription of a primary miRNA transcript (pri-miRNA) by RNA polymerases II and III. Second, the production of pri-miRNA by the nuclear ribonuclease Drosha into a precursor miRNA (pre-miRNA) with a stem-loop structure. Third, nuclear export of pre-miRNAs by exportin 5. Fourth, the cleavage of pre-miRNAs by enzyme Dicer to yield double-stranded miRNA and its helicase-mediated unwinding. Finally, the passenger strand is degraded, while the mature miRNA strand is integrated into Argonaute (AGO) protein in the RNA-induced silencing complex (RISC). miRNAs not only can mediate translational repression by interacting with mRNAs, but also target long ncRNAs (lncRNAs) and circular RNAs (circRNAs). In addition, competing endogenous RNAs (ceRNAs) can regulate other RNA transcripts by competing for shared miRNAs.

miRNAs are highly conserved, small, 21–22 nts in length, single-stranded ncRNAs, regulating a wide range of biological processes, including cell proliferation, differentiation, and apoptosis (14). The biogenesis of miRNAs is a multistep process (**Figure 1**). First, the miRNA gene is generally transcribed by RNA polymerase II and III as primary miRNA (pri-miRNA), which is dissected into 70 nts long precursor miRNA (pre-miRNA) by nuclear ribonuclease Drosha, and then exported to the cytoplasm by exportin-5 (15). Finally, the pre-miRNA is cleaved by Dicer into the double-stranded miRNA, of which the passenger strand is degraded. The mature miRNA strand is integrated into Argonaute (AGO) protein in the RNA-induced silencing complex (RISC) to mediate translational repression by targeting mRNAs. Moreover, in addition to interacting with mRNAs, miRNAs can also target lncRNAs and circRNAs, and competing endogenous RNAs (ceRNAs) manipulate other RNA transcripts by competing for shared miRNAs (16, 17).

As a novel class of endogenous ncRNAs, circRNAs were initially considered as non-functional by-products of alternative splicing (18). Subsequent reports have elucidated that circRNAs can serve as miRNAs sponges, mediate alternative splicing, and regulate the expression of parental genes (19).

**Figure 2:**
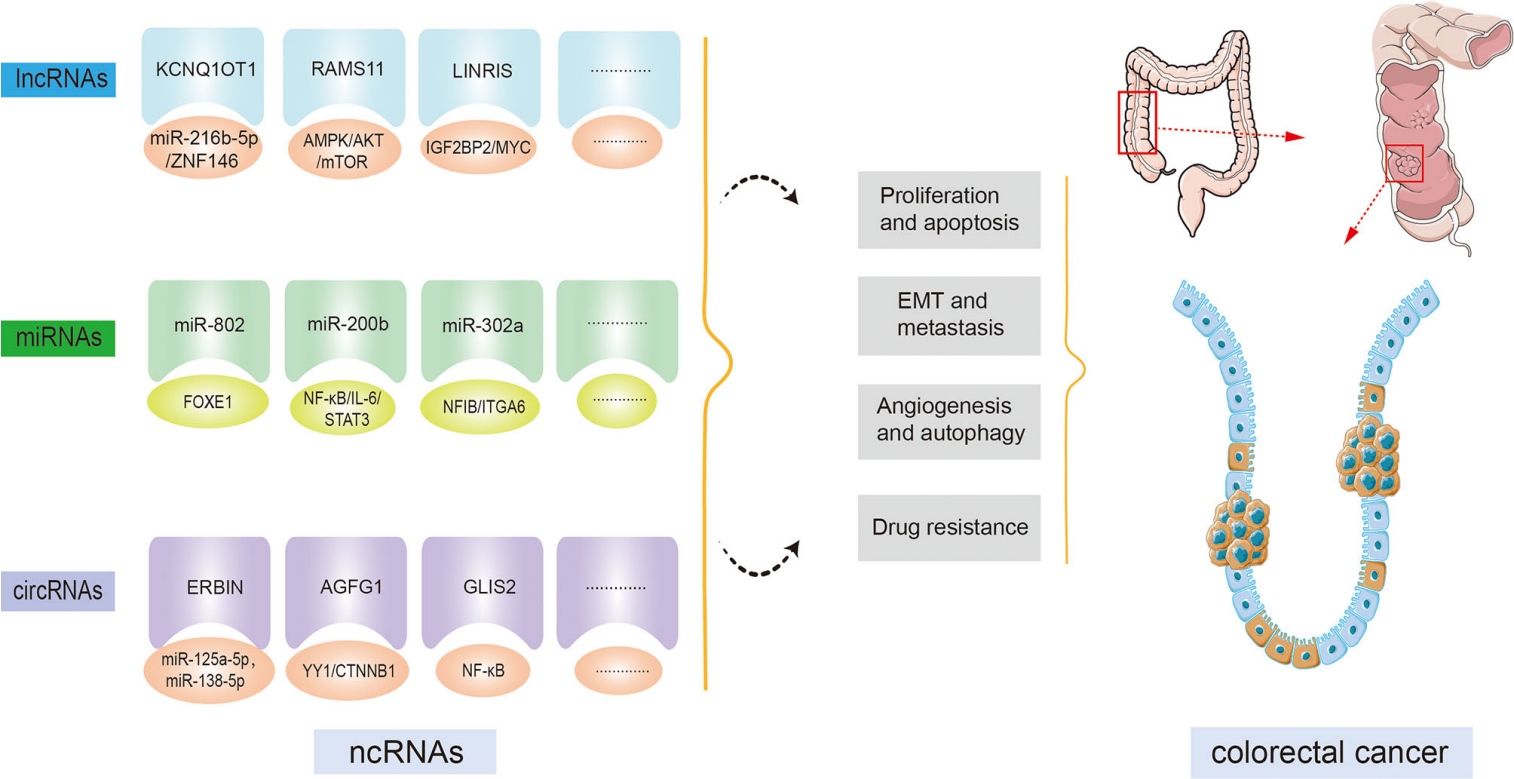
Non-coding RNAs (ncRNAs) regulatory functions in colorectal cancer (CRC) initiation and progression. By targeting downstream molecules or signaling pathways, ncRNAs are involved in CRC cell proliferation, apoptosis, epithelial-mesenchymal transition (EMT), metastasis, angiogenesis, autophagy, drug resistance, and other CRC processes.

Most recently, since the widespread implementation of tiled microarray and high-throughput sequencing techniques across the entire genome and transcript, ncRNAs have gained extensive attention as a promising tool for curing cancer. Multiple studies have uncovered that ncRNAs are involved in multiple biological processes, for example, cell proliferation, apoptosis, differentiation, and transcription (8, 20). Subsequent investigations indicate that ncRNAs are indispensable for the modulation of a variety of cancers, for example, hepatocellular carcinoma, esophageal squamous cell carcinoma, gastric cancer, and so on (21). Moreover, studies focusing on the functions of ncRNAs in CRC have increased in recent years. Numerous ncRNAs have been demonstrated to be involved in CRC development and progression (**Figure 2**). For instance, an isolated report showed that lncRNA KCNQ1OT1 was significantly overexpressed in CRC tissue. By combining with miR-216b-5p, KCNQ1OT1 could elevate the expression of ZNF146, thereby leading to an acceleration of CRC proliferation, migration, and invasion (22). Additionally, evidence is accumulating that ncRNAs can provide the possibility for exploring molecular targeted therapy and novel drug development for healing CRC patients. And these ncRNAs are relevant to the diagnosis and prognosis of CRC; therefore, the expression of ncRNAs is regarded as a regulatory factor crucial for the progression of CRC. Because the investigation of ncRNAs and cancer hallmarks, as well as tumorigenesis in CRC, has grown impressively over the last decade, making it impossible to cover each published paper. In this review, we focus on the role of prominent ncRNAs, such as lncRNAs and miRNAs, and recently emerging circRNAs in CRC development, tumorigenesis, and metastasis, as well as their clinical significance, hoping to offer a novel approach to the treatment of CRC. Other ncRNAs such as piRNAs, snoRNAs, and siRNAs will be explored in a subsequent paper.

## ncRNAs and CRC

### lncRNAs and CRC

Deregulation of lncRNA transcripts has been associated with CRC investigation to date and influences primary cancer hallmarks such as proliferation, apoptosis, metastasis, invasion, and angiogenesis (23). Additionally, lncRNAs have been associated with other biological processes such as metabolic disorders and drug resistance (**Table 1**).

**Table 1:** Selected examples of regulatory lncRNAs.

lncRNAs	Expression	Potential biomarker	Therapeutic value	Function	Target/signaling pathway	Ref
LINRIS	↑	Prognosis	NA	Promote aerobic glycolysis and autophagy while inhibiting proliferation	IGF2BP2/Myc	(24)
TCONS_00012883	↑	Prognosis, tumor size and TNM stage	NA	Promote proliferation and metastasis	DDX3/YY1/MMP1	(25)
BLACAT1	↑	Prognosis, TNM stage and distant metastasis	OXA-resistance and therapeutic target	Promote proliferation, migration, and invasion while inhibiting apoptosis	miR-519d-3p/CREB1	(26)
PiHL	↑	Prognosis	5-FU resistance and therapeutic target	Promote proliferation, and colorectal xenograft tumors	p53, GRWD1/RPL11/MDM2	(27)
ITGB8-AS1	↑	Diagnosis	Therapeutic target	Promote cell proliferation and migration	Integrin-mediated focal adhesion	(28)
AC010789.1	↑	Prognosis and lymphatic metastasis	NA	Promote proliferation, migration, invasion, and EMT	miR-432-3p/ZEB1, Wnt/β-Catenin	(29)
DNAJC3-AS1	↑	Prognosis and TNM stage	NA	Promote proliferation, migration and invasion, and lipid accumulation	EGFR/PI3K/AKT/NF-Kb/SREBP1	(30)
HOTAIR	↑	Prognosis and diagnosis	5-FU resistance and therapeutic target	Promote viability, migration, invasion and EMT	SNAIL/ HNF4α, miR-218, NF-κB/TS	(31, 32)
GAS5	↓	Diagnosis	NA	Inhibit migration and invasion while promoting apoptosis, autophagy, and AVO activities	miR-222-3p/GAS5/PTEN	(33, 34)
KCNQ1OT1	↑	Diagnosis	Cisplatin resistance and therapeutic target	Promote cell motility and proliferation while inhibit apoptosis	miR-216b-5p/ZNF14,miR-497/Bcl-2	(22, 35)
FLANC	↑	Prognosis	Therapeutic target	Promote proliferation, migration, and angiogenesis while inhibiting apoptosis	STAT3/ VEGFA	(36)
NEAT1	↑	Prognosis	NA	Promote proliferation, migration, metastasis and invasion	DDX5/Wnt/β-catenin	(37)
TPT1-AS1	↑	Prognosis TNM stage, tumor size, lymphatic metastasis, and distant metastasis	NA	Promote invasion, metastasis and angiogenesis	NF90/VEGFA	(38)
H19	↑	Prognosis	5-FU resistance and therapeutic target	Promote autophagy	SIRT1	(39)
MCF2L-AS1	↑	Prognosis	NA	Promote proliferation, invasion, and glycolysis	miR-874-3p/FOXM1	(40, 41)
RAD51-AS1	↓	Prognosis	NA	Inhibit proliferation, migration, invasion, and glycolysis	miR-29b/c-3p/NDRG2	(42)
ZNF667-AS1	↓	Prognosis	NA	Inhibit proliferation, migration, and invasion	ANK2/JAK2	(43)

↑, up-regulate; ↓, down-regulate; EMT, epithelial-mesenchymal transition; TNM, tumor node metastasis; 5-FU, 5-fluorouracil; OXA, oxaliplatin; NA, not available.

The ability of carefully selected lncRNAs to target multiple pathways that are altered in CRC makes these molecules interesting candidates for therapeutics or targets of therapeutics. HOTAIR is a well-studied lncRNA that has been widely reported as an oncogenic molecule in CRC. Previous studies had disclosed that HOTAIR knockdown inhibited cell proliferation, invasion and migration, while promoting apoptosis and enhancing cell radiosensitivity in CRC (44). A recent study showed that knockdown of HOTAIR decreased cell viability, promoted apoptosis, and inhibited cellular autophagy in CRC through upregulation of miR-93 and downregulation of autophagy-associated 12 (ATG12) (45). Furthermore, deletion of HOTAIR enhanced radiosensitivity by regulating the miR-93/ATG12 axis in CRC cells and CRC xenograft tumor models. lncRNA HOTTIP expression levels of CRC patients are identified to be elevated and intimately relative to the clinical stage and distant metastasis. Knockdown HOTTIP markedly attenuates the proliferative and migratory capability of CRC cells (46). Interestingly, another study has found that SGK1 is dramatically downregulated compared with control when HOTTIP is knocked down in HCT116 and SW620 cells. Furthermore, the decreased SKG1 can promote the expression of GSK3β and inhibit the expression of FOXO3a, which is consistent with the result of knockdown HOTTIP (47). It is worth noting that whether knockdown HPTTIP is associated with SKG1 downregulation in CRC needs further experimental verification. To prioritize all RAMS in metastatic CRC (mCRC), Silva-Fisher et al. studied whether the expression of lncRNAs is related to patient outcome (48). Notably, they found that lncRNA RAMS11 is a top upregulated lncRNA and can promote CRC growth and metastasis. And its abnormal expression also predicted unfavorable outcomes in CRC patients, suggesting the great role of RAMS11 expression as a biomarker for identifying high-risk patients (48). Another study showed that downregulation of RAMS11 significantly represses proliferation, autophagy, metastasis, and invasion of HCT-116 and SW480 cells *in vitro*. More importantly, the crucial pathway investigation suggests that Dsi-RAMS11 potentially promotes apoptosis and autophagy *via* phosphorylation of AMPK and suppression of AKT and mTOR signaling pathways (49). As a consequence, through autophagy, apoptosis and AKT/AMPKα/mTOR signaling pathways, RAMS11 downregulation is negatively associated with proliferation and metastasis of CRC cells. However, an important limitation of this study is the absence of *in vivo* demonstration, which may further confirm the conclusions. These results above support the view that cell death by lncRNA RAMS11 may occur *via* more than one regulatory mechanism and fundamental differences may exist between the AKT and mTOR pathways. Several additional lncRNAs have been implicated in CRC progression. Pharmacological screens reveal that RAMS11 enhances CRC resistance to topoisomerase inhibitors and provides mechanistic insight into the RAMS11-dependent TOP2 regulation that promotes mCRC promotion.

### miRNAs and CRC

At the beginning of the 21th century, Michael et al. found the expression of miR-143 and miR-145 were downregulated in CRC, firstly associating miRNA with CRC development (50). To date, among all types of ncRNAs, miRNAs are the best understood, which have been well studied in the occurrence and progression of CRC (5, 51). Accordingly, these molecular changes stimulate proliferation, apoptosis, metastasis, angiogenesis, and drug resistance of CRC. Particularly, the distribution of miRNAs in tumors has characteristics related to CRC diagnosis, prognosis, and response to treatment. Hence, we have summarized the functions and mechanisms of key miRNAs (**Table 2**).

**Table 2:** Selected examples of regulatory miRNAs.

miRNAs	Expression	Potential biomarker	Therapeutic value	Function	Target/signaling pathway	Ref
miR-30b-5p	↓	Liver metastasis	NA	Inhibit invasion and migration, EMT, adhesion, and motility	Rap1b	(52)
miR-133b	↓	NA	OXA and 5-FU resistance	Inhibit migration, invasion, stemness, and drug resistance	DOT1L/H3K79me2, LUCAT1/EZH2	(53, 54)
miR-450a-5p	↑	Prognosis	NA	Promote stemness and angiogenesis	SOX2	(55)
miR-25-3p	↑	Diagnosis	NA	Promote metastasis and angiogenesis	KLF2, KLF4	(56)
miR−34a	↓	Lymphatic metastasis	OXA resistance and therapeutic target	Inhibit autophagy while promoting apoptosis	SIRT1, TGF-β/Smad4	(57)
miR-106b-3p	↑	Prognosis	NA	Promoted migration, invasion, and EMT	DLC-1	(58)
miR-302a	↓	Prognosis	NA	Inhibit migration, and invasion	NFIB/ITGA6	(59)
miR-138-5p	↓	Lymphatic metastasis	Fluorouracil, doxorubicin, and cisplatin resistance	Inhibit migration and chemotherapy resistance	NFIB-Snail1	(60)
miR-146a	↓	Prognosis and liver metastasis	Cetuximab resistance and therapeutic target	Inhibit proliferation and metastasis	c-met, Snail/β-catenin	(61, 62)
miR-195b-5p	↓	Prognosis	5-FU resistance	Inhibit proliferation, migration, invasion, EMT, stemness, and M2-like TAM polarization	NOTCH2/GATA3/IL-4	(63, 64)
miR-196b-5p	↑	NA	NA	Promote proliferation, cell cycle, migration and invasion while inhibiting apoptosis	ING5	(65)
miR-214-3p	↓	Lymphatic metastasis and tumor size	NA	Inhibit proliferation and metastasis	PLAGL2/MYH9	(66)
miR-1224-5p	↓	Prognosis	NA	Inhibit metastasis, invasion and EMT	SP1-Mediated NF-κB	(67)
miR-875-3p	↓	Prognosis and distant metastasis	NA	Inhibit proliferation and migration	PLK1	(68)

↑, up-regulate; ↓, down-regulate; EMT, epithelial-mesenchymal transition; OXA, oxaliplatin; 5-FU, 5-fluorouracil; NA, not available.

miR-124, a repressive miRNA downregulated in CRC tissues, mainly through upregulating and downregulating the expression of related target genes and thus modulating molecular signaling pathways, affecting the development and treatment of CRC. On one hand, aberrantly methylated miR-124 is capable of elevating the expression of DNMT3B to promote CRC proliferation, invasion and migration (69). 5-Aza-CdR can reverse the expression level of miR-124 in Hct-116 cells by inhibiting methylation, thus reducing the expression of DNMT3B and decreasing cell proliferation, migration and invasion. On the other hand, miR-124 can target a specific region in PD-L1 3’ untranslated region (UTR) to reduce PD-L1 mRNA, protein, and cell surface expression, therefore inhibiting Tregs induction and CRC immunosuppression. As a result, the viability and proliferation of CRC cells are significantly diminished (70). Besides, miR-124 overexpression inhibits CRC proliferation and arrests the cell cycle at the G1 phase by downregulating c-Myc and induces apoptosis in CRC cells *via* upregulation of both intrinsic and extrinsic pathways. The underlying mechanism is that miR-124 downregulates Bcl-2 expression and increases the level of Bax pro-apoptotic gene, which subsequently may lead to the induction of apoptosis. miR-200 family, which modulates the expression of proteins involved in tumor metastasis and angiogenesis, is another causative collection of miRNAs downregulated in CRC. miR200 family is categorized into two groups: miR200a/b/429 and miR200c/141, which are located on chromosomes 1 and 12, respectively (71). Despite the different chromosomal locations and differences in expression patterns between the two parts, there is a significant overlap in their targets and biological functions, mainly involving components that play a role in epithelial-mesenchymal transition (EMT). And the EMT program in cancer cells is associated with an increase in cancer metastasis. Additionally, miR-200 directly inhibits the transcriptional inhibitor zinc finger E-box-binding homeobox 1, a known transcriptional suppressor of the cytoskeletal rearrangement protein E-cadherin, thus inducing EMT during CRC metastasis. Deng et al. reported that the direct target of miR-200b was the 3’-UTR of AKT2 and miR-200b induces inflammation through the AKT2-mediated NF-κB/IL-6/STAT3 signaling pathway. miR-200b regulated the expression of E-cadherin and N-cadherin that engaged in EMT (72).

EGFR (also known as Her1) is a human epidermal growth factor receptor belonging to a family of HER-related proteins that can be selectively activated by some different ligands (73). Once the ligand binds to EGFR, the receptor forms a dimer that initiates autophosphorylation of the receptor *via* tyrosine kinase activity within the receptor. Autophosphorylation triggers a series of signaling events, mainly through the RAS/RAF/MEK/MAPK and PI3K/AKT pathways (74). These pathways are responsible for cancer cell proliferation, activation of invasion and metastasis, blockade of apoptosis, and promotion of angiogenesis. Dysregulation of the EGFR pathway has been validated as a relevant procedure in CRC, and it has been identified as a target of several miRNAs. RASA1 is a member of RAS GTPase activating proteins (RAS-GAP) family (75). While the oncoprotein RAS can be inactivated by binding to the RAS-GAP members. miR-21 directly modulates RASA1 expression by targeting its 3’-UTR, and mutation or loss of function of RASA1 in CRC leads to activation of the RAS-MAPK cascade, promoting CRC progression (76). Furthermore, there is a link between EGFR and Wnt signaling. In APC-mutant CRC cells, elevated EGFR signaling boosts Wnt activity, which supports the notion that Wnt signaling is further regulated in the presence of impaired β-catenin degradation complexes. In a reciprocal manner, Wnt ligands lead to EGFR transcription through metalloproteinase-dependent binding of the cell surface EGFR ligand epitope domain to its GPCR Frizzled receptor (77). For example, miR-139-5p is a novel regulator of crosstalk between the EGFR and Wnt signaling pathways in CRC. miR-139-5p, a KRAS-responsive miRNA, is significantly downregulated in KRAS-mutated CRC tissues and cells, and its transcription is repressed by Wnt/β-catenin signaling in mutant CRC cells (78). miR-139-5p inhibits CRC cell proliferation and metastasis by targeting multiple regulators of the RAS and Wnt signaling pathways and EMT.

Nowadays, numerous lines of reports show that miRNAs serve as oncogenes or tumor suppressors in CRC evolvement (79). The critical roles of miRNAs in the pathogenesis of CRC confirm their suitability for therapeutic development.

### circRNAs and CRC

Currently, there are numerous lines of studies focusing on the mechanisms of lncRNAs and miRNAs. Compared to them, the investigation of circRNAs in the progression of human disease is still in its infancy (80, 81). Although the role of circRNAs is less well characterized in other human disorders, most studies have focused on the role of circRNAs in cancers.

ciRS-7, as a type of endogenous circRNA with a closed circular structure, was first identified by Hansen in 2011 (82). ciRS-7 plays a vital role in the transcription of RNA, the expression of downstream genes, and the synthesis of protein. Furthermore, ciRS-7 functions as an oncogene and stimulates tumor progression by competitively repressing miR-7 in various types of cancers, including CRC (82). Another study found that miR-7-mediated inhibition of the EGFR/RAF1/MAPK pathway could be alleviated by overexpression of ciRS-7 in CRC (83). Despite the mutational status of KRAS or BRAF oncogenes, miR-7 could effectively inhibit this pathway in CRC cell lines. This is due to the fact that miR-7 was able to suppress the expression of not only EGFR but also another key MAPK member, RAF1 (84). Interestingly, ciRS-7 resulted in sustained activation of the EGFR/RAF1/MAPK pathway in CRC cells regardless of treatment with low or high concentrations of miR-7 precursors. Thus, dual targeting of ciRS-7 and miR-7 could provide CRC patients with a novel therapeutic strategy to inhibit this oncogenic pathway. In addition, high ciRS-7 expression was linked to a number of clinic-pathological factors, including advanced T-stage, lymphatic and distant metastases, and consequently, patients with higher expression of ciRS-7 had a poor prognosis (83).

Most recently, an increasing number of circRNAs have been observed to be aberrantly expressed in CRC (85). More specifically, their functions in tumorigenesis and metastasis have been reported, where individual circRNAs are considered to be carcinogens or tumor suppressors. For example, Chen et al. discovered that treating RKO and HCT116 cells with cobalt chloride (Cocl2, a hypoxia mimic) or TGF-β increased circERBIN expression, implying that ERBIN may be involved in cancer progression (86). They also found that ERBIN was highly expressed in CRC cells, and the overexpression of ERBIN facilitated metastasis of CRC cells *in vitro* and *in vivo*. ERBIN exerts its oncogenic effects through regulating multiple cellular pathways, including those involved in CRC angiogenesis, proliferation, invasion, and migration. Mechanistically, ERBIN is known to directly sponge miR-125a-5p and miR-138-5p, accelerate the cap-independent protein translation of HIF-1α, and target eukaryotic translation initiation factor 4E binding protein (86).

Moreover, some studies have unveiled the great potential of circRNAs as promising prognostic markers or biomarkers in patients with CRC (87). Unlike linear RNA molecules, circRNAs possess a covalent closed-loop structure with high stability, preventing degradation induced by the exonuclease RNaseR. Furthermore, circRNAs with cell-specific or stage-specific expression patterns can be found in tissue samples, saliva, or plasma (18). These features could partially explain the possible application of circRNAs as prospective biomarkers. circ002144 expression is dramatically increased in CRC and is positively correlated with cell proliferation, migration, and invasion. The abnormal expression of circ002144 is also closely relevant to poor prognosis, which implies that circ002144 may become a promising biomarker in the prognostic evaluation of CRC (88). It is worth noting that a high level of serum circ0004771 can distinguish CRC patients from healthy individuals (89). The serum circ0004771 may become a prospective non-invasive biomarker for CRC patients. Recent progress in the circRNAs research field has unveiled central aspects of circRNA biogenesis and biology, but more needs to be known about the regulation and functions of these molecules in human disease, especially tumors. Here, we have briefly epitomized the action of important circRNAs in the progression of CRC (**Table 3**). These studies have illustrated the large diversity of strategies by which ncRNAs could modulate oncogenes or tumor suppressors to influence CRC progression.

**Table 3:** Selected examples of regulatory circRNAs.

circRNAs	Expression	Potential biomarker	Therapeutic value	Function	Target/signaling pathway	Ref
0001946	↑	Prognosis	NA	Inhibit growth, migration, and invasion while promoting EMT	miR-135a-5p/EMT	(90)
ciRS-122	↑	NA	OXA resistant and therapeutic target	Promote glycolysis and ATP production	miR-122/PKM2	(91)
100290	↑	Prognosis	NA	Promote proliferation, migration and invasion while inhibit apoptosis	miR-516b/FZD4/Wnt/β -catenin	(92)
AGFG1	↑	Liver metastasis	NA	Promote proliferation, migration, invasion and stemness while inhibiting apoptosis	YY1/CTNNB1	(93)
103809	↓	Lymphatic metastasis and clinical stage	NA	Promote proliferation and migration	miR-532-3P/FOXO4	(94)
102209	↑	Histological grade and liver metastasis	NA	Promote proliferation, migration, invasion and EMT while inhibiting cell cycle arrest and apoptosis	miR-761/RIN1	(95)
102958	↑	Prognosis and clinical stage, lymphatic metastasis	NA	Promote proliferation, migration and invasion	miR-585/CDC25B	(96)
001680		NA	Irinotecan resistance and therapeutic target	Promote proliferation, migration, and stemness	miR-340 /BMI1	(97)
0060745	↑	Lymphatic and liver metastasis, and advanced clinical stage	NA	Promote proliferation and metastasis	miR-4736/CSE1L	(98)
002144	↑	Prognosis, tumor size, lymphatic and distant metastasis, and TNM stage	NA	Promote viability, proliferation, migration, and invasion while inhibiting apoptosis	miR-615-5p/LARP1/mTOR	(88)
0000392	↑	Diagnosis, pathological stage, lymphatic and distant metastasis	NA	Promote proliferation and motility while inhibiting apoptosis	miR-193a-5p/PIK3R3/AKT	(99)
ITGA7	↓	Lymphatic metastasis, tumor size, and TNM stage	NA	Inhibit proliferation and metastasis	RAS	(100
CCDC66	↑	Hypoxia	NA	Promote viability, migration, and invasion while inhibiting apoptosis	miR−3140/autophagy	(101)
circ-133	↑	Hypoxia and TNM stage	NA	Promote metastasis	GEF-H1/RhoA	(102)
circ5615	↑	Prognosis and TNM stage	NA	Promote proliferation and cell cycle	TNKS, Wnt/β-catenin	(103)
FBXW7	↓	Tumor size	NA	Inhibit proliferation, migration and invasion	NEK2, mTOR, and PTEN	(104)
PACRGL	↑	Prognosis	NA	Promote proliferation, migration, invasion, and N1-N2 neutrophils differentiation	miR-142-3p, miR-506-3p/TGF-β1	(105)
FNDC3B	↓	Prognosis	NA	Inhibit proliferation, invasion, migration, EMT and angiogenesis	miR-97-5p/TIMP3	(106)
001971	↑	Tumor size, TNM stage, and lymphatic metastasis	NA	Promote proliferation, invasion, and angiogenesis	miR-29c-3p/ VEGFA	(107)
ZNF609	↓	Diagnosis and tumor size	NA	Promote apoptosis	p53	(108)
0026344	↓	Prognosis, diagnosis, and lymphatic metastasis	NA	Inhibit proliferation and invasion while promoting apoptosis	miR-21, miR-31	(109)
RAE1	↑	Lymphatic metastasis, and tumor size	NA	Promote migration and invasion	miR-338-3p/TYRO3	(110)

↑, up-regulate; ↓, down-regulate; EMT, epithelial-mesenchymal transition; TNM, tumor node metastasis; OXA, oxaliplatin; NA, not available.

## The Functions and Mechanisms of ncRNAs in CRC

### ncRNAs Modulate CRC Proliferation and Apoptosis

Arguably, the most fundamental feature of malignant cells involves their ability to sustain chronic proliferation (111). The proliferation process of normal cells is induced by cell cycle alternation and receptor tyrosine kinases (PTKs) activation. Interestingly, the procedure can be restricted by themselves, whereas both cell cycle checkpoints ignorance and constituent activation of RTKs failure will lead to tumorigenesis (112). At the same time, proliferation must be tolerated by tumor cells, or tumor growth will be hindered and the cells will enter senescence. Apoptosis, as a programmed cell death that occurs during the advancement or aging process of normal cells, is a kind of homeostatic mechanism to maintain the stabilization of the cell population. There are several main apoptotic pathways: the endogenous or mitochondrial pathway and the exogenous or death receptor pathway, as well as the perforin/enzyme pathway. Several of the aforementioned pathways converge at the same execution pathways or terminals, which are initiated by caspase-3 cleavage (113). Apoptosis is activated in case of extrinsic or intrinsic stressors, exerting an antitumorigenic role. In conclusion, apoptosis is a promising target for the treatment of tumors.

**Figure 3:**
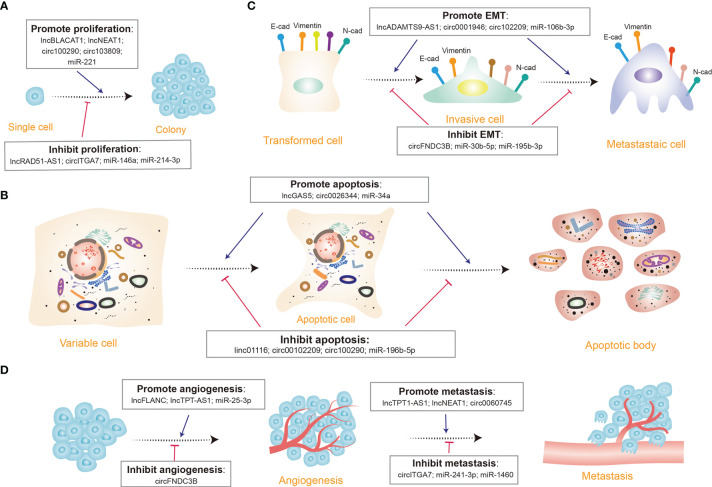
Diverse functions of non-coding RNAs (ncRNAs) in colorectal cancer. **(A)** A few ncRNAs regulate cell proliferation by promoting (e.g., lncBLACT1) or inhibiting cell proliferation progression (e.g., circITGA7). **(B)** Many ncRNAs modulate cell apoptosis *via* triggering (e.g., lncGAS5) or curbing (e.g., miR-196b-5p) cell apoptosis. **(C)** Several ncRNAs control the epithelial-mesenchymal transition (EMT) process through inducing (e.g., circ102209) or repressing (e.g., miR-30b-5p) EMT; E-cad, E-cadherin; N-cad, N-cadherin. **(D)** Certain ncRNAs manipulate tumor angiogenesis and metastasis procedure, some appear to facilitate tumor angiogenesis (e.g., lncFLANC), while others restrain (e.g., circFNDC3B) the process; some promote metastasis (e.g., lncTPT1-AS1), while others inhibit (e.g., miR-1460) the process.

Recent studies indicated that several typical ncRNAs play indispensable roles in CRC proliferation and apoptosis (**Figures 3A, B**) (94, 97). It has been identified that lncRNA SLCO4A1-AS1 is highly expressed in CRC cells, and there is a strong link between higher SLCO4A1-AS1 abundance and proliferation and apoptosis, as observed in CRC. By affecting activation of Wnt/β-catenin signaling, SLCO4A1-AS1 can mediate many cellular processes, such as cell proliferation, migration, differentiation, and apoptosis (114, 115). Consequently, elevated SLCO4A1-AS1 induces the hyper-activation of the Wnt/β-catenin signaling, thus leading to sustained proliferative ability and attenuated apoptotic behavior of CRC cells (116). Further analysis shows that SLCO4A1-AS1 knockdown severely reduced protein levels of β-catenin but not mRNA levels by the mechanism that SLCO4A1-AS1 represses phosphorylation of β-catenin, which in turn inhibits ubiquitination-mediated degradation. The more significant findings to emerge from this study is that SLCO4A1-AS1 modulated β-catenin stability by weakening the link between β-catenin and GSK3β, thereby influencing tumor progression (116). Another way that SLCO4A1-AS1 may modulate tumorigenesis is by sponging miR-508-3p, thus upregulating PARD3 and promoting CRC cell proliferation (117). Ectopic expression of circSPARC is detected in both CRC patients’ tissues and plasma. It has been unveiled that SPARC could manipulate STAT3 expression to stimulate CRC proliferation by elevating the level of c-Myc. The process mainly involves two different pathways. On the one hand, SPARC functions as a miR-485-3p decoy to enhance JAK2 activation. On the other hand, it can interact with FUS to stimulate the nuclear translocation of activated p-STAT3 (118). Another study uncovered that miR-485-3p is detected to be extensively downregulated in CRC tissues. The dysregulation of miR-485-3p is positively correlated with P21 expression and negatively correlated with TPX2 expression (119). Since the underlying mechanism has not been discussed yet, more work is needed to definitively identify the specific regulation of miR-485-3p.

It is widely believed that genetic mutations promote the uncontrolled growth and proliferation of tumor cells. However, the situation may be more complex than that. A recent study showed that there are more than 100 mutated tumor suppressor genes that do not act directly on cancer cells (120). The mechanism that drives cancer cell growth is to prevent the immune system from recognizing and destroying tumor tissue, which is the real cause of the uncontrolled spread of cancer cells. Interestingly, these mutated genes are instructing the cancer cells how to evade the immune system, rather than simply saying “grow, grow”. This may broaden our understanding of the changing traditional concept of proliferation and provide new insights for future research.

### ncRNAs Regulate CRC Metastasis

The metastatic establishment of CRC at distant organs is largely incurable and primarily contributes to the deaths of CRC patients. EMT represents the first step of metastasis and is intimately linked with the acquisition of a migratory phenotype in CRC cells (121). EMT is characterized by the loss of epithelial polarity and breakdown of tissue architecture, including the cell adhesion, the acquisition of N-cadherin and E-cadherin expression, thereby leading to the destabilized adherens junctions and increased cell mobility (122). Invasion-metastasis involves EMT and its reverse counterpart, the mesenchymal-epithelial transition, both of which are normally activated during embryonic development and tissue homeostasis, contributing to proper morphogenesis of tissues and organs (123).

Molecular underpinnings for dissemination of mCRC have been uncovered by accumulating novel paradigms in the study of mCRC (124). For instance, recent studies have illustrated the novel function of lncRNA MALAT1 in accelerating CRC metastasis through two intercellular signal transductions (125). One is MALAT1 restricting the influence of miR-15 on the transcriptional levels of LRP6 by sponging miR-15 family members, which expedites β-catenin signaling, while the other is MALAT1 binding SFPQ and interacting with the IRES domain in the 5’-UTR of the corresponding RUNX2 mRNA. As a result of both functional mechanisms, the downstream target gene RUNX2 is elevated at the transcriptional level, which is eventually linked to CRC metastasis (125). The results of another study have revealed that circAGFG1 is upregulated with a significant increase in CRC metastasis (93). circAGFG1 directly binds to miR-4262 and miR-185-5p to enhance YY1 expression and CTNNB1 transcription, contributing to an acceleration of CRC metastasis (93). Consistently, the expression of lncRNA H19 is higher in mesenchymal subtypes of CRC cells than in epithelial subtypes. In CRC, H19 is found to be specifically overexpressed clones capable of seeding metastases, while short hairpin RNA (shRNA)-mediated knockdown of H19 in metastasis-capable clones abrogates the development of distant metastases (126). Based on this study, H19 acts as a ceRNA against miR-200b and miR-200c, resulting in derepression of ZEB1 and GIT2 in primary tumors and distant metastases. Nonetheless, GIT2 is shown to facilitate colonization at metastatic sites (127). An important strength of this study is relying on endogenous AGO pulldown, which provides biochemical support rather than nucleic acid sequence analysis or indirect measures of ceRNA activity, as well as targeted mutagenesis to verify the feature of H19 as ceRNA, despite the controversy regarding ceRNAs in general. Furthermore, miRNAs also exert an important role during CRC metastasis. Compared to normal tissues, the expression of miR-302a is substantially decreased in CRC tissues, especially in patient-derived xenografts and CTX-resistant cells. miR-302a overexpression inhibits metastasis in CRC cells and restores CTX responsiveness (59). miR-302a has also been observed to repress metastasis-promoting effect of NFIB that physiologically stimulates ITGA6 transcription. By decreasing CD44-induced cancer stem cell-like properties, EGFR-mediated MAPK, and AKT activation, miR-302a restores CTX responsiveness. Many ncRNAs have been demonstrated to be involved in EMT and metastasis of CRC (**Figures 3C, D**). Emerging evidence has depicted the specific functions and mechanisms of ncRNAs in CRC cells during their metastatic journey, which may be exploited to possibly halt metastatic growth and even prevent successful dissemination. Additionally, it would be interesting to see how these ncRNAs function during other steps in the invasion-metastasis cascade. Future work will be designed to substantiate the mechanisms of these ncRNAs in contributing to EMT and metastasis and to find potential novel therapeutic approaches for the diagnosis and treatment of CRC.

### ncRNAs Manipulate CRC Angiogenesis

Despite their physiological and morphological homogeneity, tumors display a wide range of morphological and phenotypic differences, including the expression of receptors on the surface of cells and their angiogenic capabilities (128). Angiogenesis is identified as an important hallmark of malignant tumors because quantities of blood vessels are needed to feed cancer cells (129). A number of ncRNAs have recently been demonstrated to be auxiliary diagnostic biomarkers for a range of cancers, and their expression varies among them. Likewise, the most recent research indicated that ncRNAs were heterogeneous in their regulation of tumor angiogenesis.

There are some frequently dysregulated ncRNAs involved in angiogenesis (**Figure 3D**). In clinical samples, miR-450a-5p is negatively correlated with SOX2 (55). An isolated study has discovered that miR-450a-5p induces angiogenesis *via* directly targeting the 3’-UTR regions of SOX2 in CRC (130). In addition, many miRNAs are packaged within tumor cell-derived exosomes, emerging as vital contributors to the complicated modulation and balance of pro-and anti-angiogenic molecules. Exosomal miR-25-3p-derived from CRC cells has been shown to be associated with angiogenesis (56). Exosomal miR-25-3p disrupts endothelial barrier integrity, increases vascular permeability, and simultaneously triggers angiogenesis. circ001971 expression is dramatically elevated in CRC tissues. Knockdown of circ001971 in CRC cells significantly interrupts HUVEC tube formation, consequently reducing the angiogenesis and tumor growth of SW620 cell-derived cancer *in vitro* (107).

VEGFA, a member of the growth factor family, has a strong capacity to activate the angiogenic milieu by increasing microvascular density and vascular permeability, which promotes tumor angiogenesis and metastasis and leads to tumors resistant to antiangiogenic therapy. The Krüppel-like factor (KLF) factor family is composed of zinc finger-containing transcription factors that regulate a variety of biological processes (131). miR-25-3p is recently been demonstrated to induce vascular permeability and angiogenesis in vascular endothelial cells by downregulating KLF2 and KLF4. Additionally, both KLF2 and KLF4 are downregulated in CRC (56). Specifically, KLF2 negatively regulates angiogenesis by inhibiting VEGFR221, whereas KLF4 maintains the integrity of endothelial barrier function by promoting tight junction-related proteins including ZO-1 and ocludin5 (132). Furthermore, cir001971 acts as a ceRNA to relieve miR-29c-3p-induced VEGFA inhibition, which contributes to the aggravation of angiogenesis in CRC (107). An additional study indicated that lncRNA FLANC was involved in CRC angiogenesis *via* the STAT3/VEGFA pathway (36). Western-blot data confirmed that the overexpression of FLANC leads to a higher level of the phosphorylated STAT3, which acts as the active form triggering VEGFA (36). In conclusion, these findings lead to the determination that different ncRNAs exert multifaceted and even contradictory roles in regulating CRC angiogenesis through several distinct mechanisms, thereafter proving the heterogeneity of ncRNAs. Hence, we speculate that future research will focus on angiogenesis inhibitors and, how ncRNAs serve their roles in effective targeted therapies to repress angiogenesis will be studies focus.

### ncRNAs Regulate CRC Autophagy

Autophagy is a cellular catabolic and evolutionarily conserved process, which removes redundant or dysfunctional components by dissociating the defective or malfunctioning organelles inside the cells (133, 134). Autophagy has a complex and context-dependent role in carcinogenesis. In addition to removing damaged organelles and aggregated proteins, autophagy serves as a surveillance mechanism to protect normal cells from the transformation into cancerous cells by reducing DNA damage, reactive oxygen species, and damaged mitochondria (135). Multiple lines of evidence have established that ncRNAs, as important regulatory molecules in CRC, are also involved in the autophagy of tumor cells (136). Once cancer occurs, autophagy is upregulated to ensure the survival of tumor cells in crisis circumstances such as hypoxia and growth factor deprivation. Inhibition or promotion of ncRNAs could decrease autophagy, allowing therapeutic strategies and treatment agents more effective (137). Through their ability to influence autophagy in CRC cells, miRNAs play a crucial role in the occurrence and development of CRC. The precise processes through which miRNAs influence autophagy and regulate cancer occurrence and progression have not been established. Inhibition of protective autophagy by miR-27b-3p may lead to increased sensitivity of CRC cells to chemotherapy, as ectopic expression of miR-27b-3p is downregulated in oxaliplatin-resistant CRC cells (HCT116-OxRS and W480-OxR) versus the corresponding parental cells. Impairing autophagy in CRC cells by reducing ATG10 expression can make them more susceptible to chemotherapeutic agents *in vitro* and *in vivo*, which is consistent with the report that miR-27b-3p levels positively correlate with disease-free survival time in CRC patients (138). Besides, lncRNAs also affect autophagy by modulating the expression of ATG genes, which are key regulators in the autophagy process (139). HOTAIR-mediated autophagy was reported to be a crucial event in the development and progression of CRC. As a molecular sponge of miR-93, lncRNA HOTAIR could modulate the expression of ATG12 to induce CRC autophagy (45). Through downregulation of miR-34a, lncRNA NEAT1 promoted autophagy in CRC cell lines. An in-depth study of its molecular mechanism revealed that NEAT1 targeted miR-34a, while miR-34a was shown to target putative binding sites in the 3’-UTR, ATG9A, and ATG4B of HMGB1, which were involved in autophagy activation (140). And inhibition of the putative targets of miR-34a involved in autophagy revealed a new pathway for NEAT1 to regulate chemoresistance. Moreover, a recent study revealed that dysregulation of lncRNA GAS5 expression could induce autophagy in CRC (33). GAS5 facilitated the formation of vesicle-like structures and autolysosome structures in cells. lncRNAs frequently function as ceRNAs for modulating autophagy-related miRNAs. A detailed cellular mechanism explained that GAS5 enhanced PTEN expression by acting as a ceRNA of miR-222-3p, which subsequently promoted CRC cell autophagy (33). It was reported that modulation of miR-20a was substantially lower under hypoxia that facilitated hypoxia-induced autophagy in CRC cells (141). Also, research showed that the ectopic expression of circCCDC66 could interact with miR-3140 in autophagy (101).

Given that aberrant autophagy is implicated in the pathogenesis of CRC, deliberate manipulation of autophagy may be effective in treating CRC. A precise determination of autophagy activity is essential to downstream autophagy-based therapy for CRC since autophagy dynamics vary greatly within different cellular statuses, and both an increase and a decrease in autophagy can contribute to CRC pathogenesis. Consequently, the importance of tailoring interventions with autophagy regulators in CRC of particular situations rather than applying a ‘one size fits all’ approach is vital for favorable treatment outcomes.

## The Clinical Significance of ncRNAs in CRC

The clinical significance of ncRNAs in CRC explains their capability for diagnosis, treatment, chemotherapeutic resistance, and prognosis. To date, the diagnosis of CRC is mainly dependent on colonoscopy, which is deemed as the gold standard for the diagnosis of CRC (142). Furthermore, this approach has the disadvantage of requiring intestinal preparation and the risk of intestinal rupture, and it is not suitable for patients with anorectal stenosis, peritoneal irritation, severe cardiopulmonary function, and other conditions (143). Therefore, novel biomarkers for diagnosis and effective therapeutic targets are urgently required to improve the current situation.

### ncRNAs as Potential Biomarkers for CRC Diagnosis and Prognosis

A competent biomarker should be sensitive, specific, repeatable, stable, and useful in clinical settings. ncRNAs are attractive candidates for biomarkers due to their expression patterns and properties (universality, conservation, tissue/cell selectivity, and stability). Besides, ncRNAs are enriched in human bodily fluids, such as plasma and saliva, facilitating their detection and making them suitable markers for cancer detection, particularly in liquid biopsies. These studies suggest that ncRNAs can serve as biomarkers for improving current detection and diagnostic methods of CRC. The abnormal expression of ncRNAs in CRC should be the prerequisite for them as biomarkers in clinical practice. Furthermore, ncRNAs as a non-invasive technology thereby being an ideal diagnostic approach have attracted much attention. Research has shown that miR-29a and miR-224 could be informative biomarkers for screening and early diagnosis of CRC *via* a noninvasive way (144). Survival of patients with metastatic cancer is still unfavorable, and resistance to therapy remains a major barrier to effective treatment. One intriguing approach to improving cancer treatment is leveraging cancer- and tissue-specific expression profiles to develop prognostic and diagnostic markers for the progression from primary to metastatic diseases.

Ling et al. found that patients with higher miR-224 levels appeared to have unfavorable overall survival in the five CRC cohorts (145). More interestingly, the incorporated analysis with CDH1 dramatically enhanced the predictive power of miR-224 for survival evaluation instead of appraising miR-224 alone (145). In addition to the diagnostic value of miR-224, it might also become a potential prognostic marker in CRC. Another research discovered that miR-224 mediates CRC tumorigenesis through regulating Wnt/β-catenin signaling, further proving that miR-224 was a prognostic biomarker (146). Assessment of miR-203 in serum could be an attractive and promising clinical tool for identifying patients with mCRC. Serum miR-203 was significantly correlated with the metastatic phenotype of CRC and was an independent prognostic biomarker for liver, lymph node, and peritoneal metastases of CRC, respectively (147). Moreover, serum miR-203 levels were significantly upregulated in a stage-dependent manner, and higher miR-203 expression was associated with poor survival in CRC patients, which suggested that miR-203 in serum was an independent prognostic marker.

A recent study illustrated that circ0004771 expression was exceptionally up-regulated in CRC patients, implying circ0004771 could provide a novel biomarker for the diagnosis of CRC (89). Recently, evidence is accumulating that ncRNAs can be not only clinical biomarkers for the early diagnosis and detection, as well as prognosis of cancer, but also potential therapeutic targets to enhance anti-tumor responses by regulating ncRNAs.

### ncRNAs as Promising CRC Therapeutic Targets

Chemotherapy, radiotherapy, and excision are the main therapeutic strategies for clinical CRC (148). Although the clinical treatment has been improved, patients tend to have an unfavorable prognosis, and their 5-year survival rates remain low, which seems to be relevant to the chemotherapeutic resistance of CRC cells (149). Of note, recent studies have shown that ncRNAs can affect chemo-resistance in CRC therapy (150, 151). Therefore, identifying novel therapeutic targets for optimizing CRC therapy can be achieved through understanding the regulatory mechanisms of ncRNAs involved in chemotherapy and radiotherapy resistance.

On the one hand, it is well known that 5-Fu is a commonly used chemotherapeutic agent in curing CRC, and it obstructs DNA replication by inhibiting thymidylate synthase, hence leading to cell cycle arrest and apoptosis to induce DNA damage (152). lncRNA H19 level is found to increase in CRC, and it strongly enhances the resistance of CRC cells to 5-Fu. Moreover, molecular mechanism suggests that H19 induces chemotherapy resistance by binding to the downstream target gene SIRT1 (39). On the other hand, cancer stem cells (CSC), as one of the subpopulations of chemo-resistant cells, play a crucial role in disease recurrence after chemotherapy. miR-133b is rich in CSCs and associated with stem cell-like characteristics, mainly elevated surface markers of CSCs and enhanced chemotherapeutic resistance (53). Furthermore, miR-133b overexpression reduces stem cell gene DOT1L-mediated H3K79me2 modification, which is consistent with down-regulation of stem cell gene transcription (53). Recovery of DOT1L eliminates the inhibitory effect of miR-133b on stem cell and CRC chemoresistance (54). miR-27a is observed to be overexpressed in the progress of anti-therapy of CRC. More importantly, miR-27a can act as a key regulatory factor engaging in metabolism reprogramming that might stimulate the mechanism concerning the chemical resistance of CRC (153).

The features of ncRNAs in CRC elucidate they act as promising treatment candidates for new therapeutic interventions. It is noticeable that circRNA PTK2 interacting with vimentin exerts a catalytic role during CRC growth and metastasis (154). In addition, vein injection of shRNA distinctively targeting circPTK2 immensely blunts CRC metastasis among the patient-derived xenograft models (154). Therefore, circPTK2 might afford an unrealized therapeutic target for the remedy of CRC metastasis. The ability to manipulate ncRNA expression and activity *in vivo* through anti-ncRNAs or ncRNA mimics provides an opportunity for developing innovative therapeutic approaches to CRC. miRNA dysregulation is causal in many cancer cases. miRNA mimics and molecules capable of targeting miRNAs have shown promise in preclinical development. Numerous strategies have been investigated to complement miRNAs with tumor suppressor functions by using miRNA mimics, which are synthetic oligonucleotide duplexes that mimic the function of their naturally occurring miRNA counterparts. Such miRNA mimics can be chemically modified to be more stable or capable of targeted delivery to tumors. miR−34a mimics can be encapsulated into TCP1-CD-QD nanoparticles and transferred into CRC cells, which contributes to the suppression of the proliferation and migration of CRC cells *in vitro* and inhibition of tumor growth in a tumor xenograft model derived from CRC cells (155). Moreover, the obtained indicates that co-delivery of miR-34a mimics and 5-FU could achieve synergistic effects for CRC treatment.

Although only a few typical ncRNAs related to chemoresistance are included in this review, more ncRNAs regulating CRC radiotherapy and chemoresistance need to be further explored. On the other hand, explaining the underlying capacities of ncRNAs in predicting treatment responses and managing individualized treatment choices appears to be another interesting aspect. Particularly, the combination of ncRNAs and chemotherapeutic agents to sensitize CRC seems promising and worthy of clinical trials. Future studies focusing on the regulation of ncRNAs in CRC chemo-resistance may contribute to certifying ncRNAs as encouraging therapeutic candidates. With the increasing involvement of ncRNAs revealed by the studies of chemoradiation resistance in CRC cells, as novel biomarkers, ncRNAs have great potential not only for predicting the efficiency of chemoradiation and prognosis, but for interfering with chemo-radiation resistance as targets in clinical CRC therapies.

### Other Clinical Significance of ncRNAs in CRC

In addition to being potentially applied as diagnostic or prognostic biomarkers and therapeutic targets of CRC, the dysregulation of ncRNAs is correlated with the TNM stage, lymphatic metastasis, tumor size, and differentiation grade. For example, circ103809 participates in TNM staging of CRC and can be used as a biomarker (94). The expression of lncRNA DANCR is highly upregulated in CRC, which is associated with the TNM stage (156). Statistical analysis has demonstrated that lncRNA RPPH1 is positively associated with advanced TNM (157). Furthermore, *in situ* hybridization showed that high expression of RPPH1 in advanced TNM stage was associated with poor metastasis and overall survival (157). Overexpression of circCAMSAP1 is tightly linked with stage and clinical stage (158). Zhang et al. have discovered that lncRNA CASC11 expression is positively associated with tumor size, lymph node metastasis, and TNM stage in CRC (159). These ncRNAs mentioned above can provide a basis for the diagnosis and grading of clinical diseases and clues for predicting the prognostic outcomes.

## Conclusion and Prospect

CRC-related ncRNAs are increasingly becoming one of the most scorching topics in RNA biology and oncology. To date, lncRNAs, miRNAs, and circRNAs are the most commonly investigated ncRNAs that are involved in CRC progression. Of note, the functions and mechanisms of other types of ncRNAs are still unclear but are currently emerging. For instance, it has been revealed that piRNA-54265 is extensively upregulated in CRC and the elevated expression of piRNA-54265 in tumor or serum is significantly correlated with an unfavorable prognosis. Functional assays have revealed that piRNA-54265 targets PIWIL2 protein and this is essential for the formation of PIWIL2/STAT3/phosphorylated-SRC complex, which facilitates STAT3 signaling and enhances proliferation, metastasis as well as chemo-resistance of CRC cells (160). Several recent studies have independently unveiled that snoRNAs are involved in various cancers progression. SNORD126 is observed to be upregulated in CRC clinical samples, and it can promote tumor growth by modulating the PI3K/AKT signaling pathway rather than functioning as a miRNA (161).

The tRNA-derived fragments (tRFs) are generated through endo-nucleolytic cleavage of corresponding tRNAs and have been shown to be potential biomarkers for tumor diagnosis and treatment in several studies (162). For example, tRF levels in breast cancer cells significantly increased under hypoxia, which is consistent with their potential roles in stress response. Goodarzi et al. found that endogenous tRFs destabilize oncogenic transcripts by binding directly to YBX1 (163). Overexpression of YBX1 is associated with tumorigenic phenotypes and has been shown to facilitate cancer metastasis (164). Furthermore, there is a high correlation between increased expression of multiple tRF-YBX1 targets (EIF4G1, EIF4EBP1 and EIF3B) and reduced recurrence-free survival. Transcripts of these oncogenes function in various aspects of cellular function, including translation and cellular signaling, and are repressed by tRFs in breast cancer cells (163). Recently, studies on the impact of CRC-related tRFs or key tRFs on CRC progression and related mechanisms are emerging. After hypoxic treatment, a total of 14 tRFs were differentially expressed in hypoxia-induced CRC RKO cells by performing tRF sequencing and real-time PCR assays. Among them, tRF-20-M0NK5Y93 might be a promising target for exploration, as its expression was significantly lower under hypoxic conditions than control conditions, and tRF-20-M0NK5Y93 inhibited CRC cell invasion and migration by targeting the EMT-related molecule Claudin-1 (165). Another study showed that tRF/miR-1280, a 17 bp fragment derived from tRNA^Leu^ and pre-miRNA, was low expression in CRC specimens. Mechanistic investigations indicated that the Notch ligand JAG2 was a direct target of tRF/miR-1280 binding and that tRF/miR-1280 inhibited colorectal cancer growth and metastasis by suppressing the Notch signaling pathway that supported the CSC phenotype (166). As for the other ncRNAs, such as eRNAs and paRNAs, no study has reported any strong connections between the ectopic expression of these ncRNAs and human cancers.

In recent years, a limited number of proteins or peptides encoded by ncRNAs have been demonstrated to exhibit significant biological and pathological functions in the tumorigenesis and progression of CRC. For example, it has been reported that lncRNA HOXB-AS3 encodes a conserved 53-aa peptide that inhibits CRC growth by regulating the reprogramming of tumor metabolism and alternative splicing of pyruvate kinase (167). Additionally, function experiments have revealed that circMAPK14-175aa (a 175 amino acid peptide) encoded by circRNA MAPK14 can suppress the CRC malignant phenotype, thus affecting CRC progression and metastasis (168). Currently, some lncRNAs and circRNAs have been shown to encode proteins or peptides, and studies on the coding functions of miRNAs are emerging. A protein and a peptide (miPEP-200a and miPEP-200b) encoded by pri-miRNA (miR-200a and miR-200b) suppress the migration of prostate cancer cells by inhibiting the EMT process (8). These findings broaden the understanding of ncRNA and provide further insight into the function of ncRNAs.

The intestinal microbiota, composed of a considerable population of microorganisms, is maintained by dynamic host-microbiota interactions (169). Numerous studies have shown a link between intestinal dysbiosis and CRC (170). The roles of intestinal microorganisms in initiating and facilitating the CRC process are being increasingly understood. However, few studies focus on ncRNAs in the modulation of dynamic host-microbiota interactions, and the molecular regulators of ncRNAs in intestinal microbiota are still not fully understood. The existence of thousands of ncRNAs involved in the intracellular network regulation obtains essential implications for our understanding of CRC, which in turn forces us to develop our unique view of the disorder, from its causative origins to available treatment options and additional treatment strategies.

The contribution of ncRNAs in the genesis and progression of human illnesses is gaining popularity, but more research is needed to identify the entire extent of this contribution and the processes by which ncRNAs exert their pathological effects. Consequently, what comes to the first is a more pronounced understanding of ncRNAs function and mechanisms, both in CRC physiological and pathological conditions. In this review, we have summarized the latest research on ncRNAs that operate as promotors or tumor suppressors participating in CRC proliferation, apoptosis, invasion, metastasis, angiogenesis, autophagy, and chemo-resistance. Recent research has improved our conception of ncRNAs: not only do they perform fundamental operations in the normal physiological management processes, but also take part in abnormal pathologic regulatory processes. Although the research on ncRNAs has made great progress in recent years, the molecular mechanisms of administering aspects in CRC are still not clear, and further detailed mechanism research is urgently needed. Therefore, more pioneering studies are required for further exploration of the diagnostic and therapeutic opportunities that ncRNAs offer.

## Author Contributions

FZ collected the related paper. ZJ wrote the draft and revised it. JA and ZL collected the tables and designed them. All authors contributed to the article and approved the submitted version.

## Conflict of Interest

The authors declare that the research was conducted in the absence of any commercial or financial relationships that could be construed as a potential conflict of interest.

## Publisher’s Note

All claims expressed in this article are solely those of the authors and do not necessarily represent those of their affiliated organizations, or those of the publisher, the editors and the reviewers. Any product that may be evaluated in this article, or claim that may be made by its manufacturer, is not guaranteed or endorsed by the publisher.

## References

[1] Yamashita R Long J Longacre T Peng L Berry G Martin B. Deep Learning Model for the Prediction of Microsatellite Instability in Colorectal Cancer: A Diagnostic Study. Lancet Oncol (2021) 22(1):132–41. doi: 10.1016/S1470-2045(20)30535-0 33387492

[2] Dekker E Tanis PJ Vleugels JLA Kasi PM Wallace MB. Colorectal Cancer. Lancet (London England) (2019) 394(10207):1467–80. doi: 10.1016/S0140-6736(19)32319-0 31631858

[3] Muzny DM Bainbridge MN Chang K Dinh HH Drummond JA Fowler G. Comprehensive Molecular Characterization of Human Colon and Rectal Cancer. Nature (2012) 487(7407):330–7. doi: 10.1038/nature11252 PMC3401966 22810696

[4] Zhou XY Luo B Jiang ZK Xie YK Wu FC Huang JQ. Non-Coding RNAS and Colorectal Cancer Liver Metastasis. Mol Cell Biochem (2020) 475(1-2):151–9. doi: 10.1007/s11010-020-03867-8 32767228

[5] Balacescu O Sur D Cainap C Visan S Cruceriu D Manzat-Saplacan R. The Impact of miRNA in Colorectal Cancer Progression and Its Liver Metastases. Int J Mol Sci (2018) 19(12). doi: 10.3390/ijms19123711 PMC6321452 30469518

[6] Ogunwobi OO Mahmood F Akingboye A. Biomarkers in Colorectal Cancer: Current Research and Future Prospects. Int J Mol Sci (2020) 21(15). doi: 10.3390/ijms21155311 PMC7432436 32726923

[7] Anastasiadou E Jacob LS Slack FJ. Non-Coding RNA Networks in Cancer. Nat Rev Cancer (2018) 18(1):5–18. doi: 10.1038/nrc.2017.99 29170536 PMC6337726

[8] Wang J Zhu S Meng N He Y Lu R Yan GR. ncRNA-Encoded Peptides or Proteins and Cancer. Mol Ther (2019) 27(10):1718–25. doi: 10.1016/j.ymthe.2019.09.001 PMC6822234 31526596

[9] Lei B Tian Z Fan W Ni B. Circular RNA: A Novel Biomarker and Therapeutic Target for Human Cancers. Int J Med Sci (2019) 16(2):292–301. doi: 10.7150/ijms.28047 30745810 PMC6367529

[10] Statello L Guo C-J Chen L-L Huarte M. Gene Regulation by Long Non-Coding RNAs and Its Biological Functions. Nat Rev Mol Cell Biol (2021) 22(2):96–118. doi: 10.1038/s41580-020-00315-9 33353982 PMC7754182

[11] Winkle M El-Daly SM Fabbri M Calin GA. Noncoding RNA Therapeutics - Challenges and Potential Solutions. Nat Rev Drug Discov (2021) 20(8):629–51. doi: 10.1038/s41573-021-00219-z PMC8212082 34145432

[12] Peng WX Koirala P Mo YY. LncRNA-Mediated Regulation of Cell Signaling in Cancer. Oncogene (2017) 36(41):5661–7. doi: 10.1038/onc.2017.184 PMC6450570 28604750

[13] Rinn JL Chang HY. Genome Regulation by Long Noncoding RNAs. Annu Rev Biochem (2012) 81:145–66. doi: 10.1146/annurev-biochem-051410-092902 PMC3858397 22663078

[14] Chen L Heikkinen L Wang C Yang Y Sun H Wong G. Trends in the Development of miRNA Bioinformatics Tools. Brief Bioinform (2019) 20(5):1836–52. doi: 10.1093/bib/bby054 PMC7414524 29982332

[15] Liu B Li J Cairns MJ. Identifying miRNAs, Targets and Functions. Brief Bioinform (2014) 15(1):1–19. doi: 10.1093/bib/bbs075 23175680 PMC3896928

[16] Tay Y Rinn J Pandolfi PP. The Multilayered Complexity of ceRNA Crosstalk and Competition. Nature (2014) 505(7483):344–52. doi: 10.1038/nature12986 PMC4113481 24429633

[17] Salmena L Poliseno L Tay Y Kats L Pandolfi PP. A ceRNA Hypothesis: The Rosetta Stone of a Hidden RNA Language? Cell (2011) 146(3):353–8. doi: 10.1016/j.cell.2011.07.014 PMC3235919 21802130

[18] Ma Z Shuai Y Gao X Wen X Ji J. Circular RNAs in the Tumour Microenvironment. Mol Cancer (2020) 19(1):8. doi: 10.1186/s12943-019-1113-0 31937318 PMC6958568

[19] Yin Y Long J He Q Li Y Liao Y He P. Emerging Roles of circRNA in Formation and Progression of Cancer. J Cancer (2019) 10(21):5015–21. doi: 10.7150/jca.30828 PMC6775606 31602252

[20] Esmaeili M Keshani M Vakilian M Esmaeili M Peymani M Seyed Forootan F. Role of non-Coding RNAs as Novel Biomarkers for Detection of Colorectal Cancer Progression Through Interaction With the Cell Signaling Pathways. Gene (2020) 753:144796. doi: 10.1016/j.gene.2020.144796 32450203

[21] Dragomir MP Kopetz S Ajani JA Calin GA. Non-Coding RNAs in GI Cancers: From Cancer Hallmarks to Clinical Utility. Gut (2020) 69(4):748–63. doi: 10.1136/gutjnl-2019-318279 32034004

[22] Zhu S Chen CY Hao Y. LncRNA KCNQ1OT1 Acts as miR-216b-5p Sponge to Promote Colorectal Cancer Progression *via* Up-Regulating ZNF146. J Mol Histol (2021) 52:479–90. doi: 10.1007/s10735-020-09942-0 33394291

[23] Poursheikhani A Abbaszadegan MR Kerachian MA. Mechanisms of Long non-Coding RNA Function in Colorectal Cancer Tumorigenesis. Asia Pac J Clin Oncol (2020) 17:7–23. doi: 10.1111/ajco.13452 32970938

[24] Wang Y Lu J-H Wu Q-N Jin Y Wang D-S Chen Y-X. LncRNA LINRIS Stabilizes IGF2BP2 and Promotes the Aerobic Glycolysis in Colorectal Cancer. Mol Cancer (2019) 18(1):174. doi: 10.1186/s12943-019-1105-0 31791342 PMC6886219

[25] Yang P Li J Peng C Tan Y Chen R Peng W. TCONS_00012883 Promotes Proliferation and Metastasis *via* DDX3/YY1/MMP1/PI3K-AKT Axis in Colorectal Cancer. Clin Trans Med (2020) 10(6):e211. doi: 10.1002/ctm2.211 PMC7568852 33135346

[26] Chen R Zhou S Chen J Lin S Ye F Jiang P. LncRNA BLACAT1/miR-519d-3p/CREB1 Axis Mediates Proliferation, Apoptosis, Migration, Invasion, and Drug-Resistance in Colorectal Cancer Progression. Cancer Manag Res (2020) 12:13137–48. doi: 10.2147/CMAR.S274447 PMC7764561 33376405

[27] Deng X Li S Kong F Ruan H Xu X Zhang X. Long Noncoding RNA PiHL Regulates P53 Protein Stability Through GRWD1/RPL11/MDM2 Axis in Colorectal Cancer. Theranostics (2020) 10(1):265–80. doi: 10.7150/thno.36045 PMC6929633 31903119

[28] Lin X Zhuang S Chen X Du J Zhong L Ding J. lncRNA ITGB8-AS1 Functions as a ceRNA to Promote Colorectal Cancer Growth and Migration Through Integrin-Mediated Focal Adhesion Signaling. Mol Ther J Am Soc Gene Ther (2021) 30. doi: 10.1016/j.ymthe.2021.08.011 PMC8821934 34371180

[29] Duan W Kong X Li J Li P Zhao Y Liu T. LncRNA AC010789.1 Promotes Colorectal Cancer Progression by Targeting MicroRNA-432-3p/ZEB1 Axis and the Wnt/beta-Catenin Signaling Pathway. Front Cell Dev Biol (2020) 8:565355. doi: 10.3389/fcell.2020.565355 33178684 PMC7593606

[30] Tang Y Tang R Tang M Huang P Liao Z Zhou J. LncRNA DNAJC3-AS1 Regulates Fatty Acid Synthase *via* the EGFR Pathway to Promote the Progression of Colorectal Cancer. Front Oncol (2020) 10:604534. doi: 10.3389/fonc.2020.604534 33604287 PMC7885865

[31] Jin L Pan YL Zhang J Cao PG. LncRNA HOTAIR Recruits SNAIL to Inhibit the Transcription of HNF4alpha and Promote the Viability, Migration, Invasion and EMT of Colorectal Cancer. Transl Oncol (2021) 14(4):101036. doi: 10.1016/j.tranon.2021.101036 33588137 PMC7901038

[32] Li P Zhang X Wang L Du L Yang Y Liu T. lncRNA HOTAIR Contributes to 5FU Resistance Through Suppressing miR-218 and Activating NF-κb/TS Signaling in Colorectal Cancer. Mol Ther Nucleic Acids (2017) 8:356–69. doi: 10.1016/j.omtn.2017.07.007 PMC5537205 28918035

[33] Liu L Wang H-J Meng T Lei C Yang X-H Wang Q-S. lncRNA GAS5 Inhibits Cell Migration and Invasion and Promotes Autophagy by Targeting miR-222-3p *via* the GAS5/PTEN-Signaling Pathway in CRC. Mol Ther Nucleic Acids (2019) 17:644–56. doi: 10.1016/j.omtn.2019.06.009 PMC6698928 31400607

[34] Liu L Meng T Yang X-H Sayim P Lei C Jin B. Prognostic and Predictive Value of Long non-Coding RNA GAS5 and mircoRNA-221 in Colorectal Cancer and Their Effects on Colorectal Cancer Cell Proliferation, Migration and Invasion. Cancer Biomark (2018) 22(2):283–99. doi: 10.3233/CBM-171011 PMC13078424 29630521

[35] Zheng ZH You HY Feng YJ Zhang ZT. LncRNA KCNQ1OT1 is a Key Factor in the Reversal Effect of Curcumin on Cisplatin Resistance in the Colorectal Cancer Cells. Mol Cell Biochem (2020) 476:2575–85. doi: 10.1007/s11010-020-03856-x 32757174

[36] Pichler M Rodriguez-Aguayo C Nam SY Dragomir MP Bayraktar R Anfossi S. Therapeutic Potential of FLANC, a Novel Primate-Specific Long non-Coding RNA in Colorectal Cancer. Gut (2020) 69(10):1818–31. doi: 10.1136/gutjnl-2019-318903 PMC7382985 31988194

[37] Zhang M Weng W Zhang Q Wu Y Ni S Tan C. The lncRNA NEAT1 Activates Wnt/β-Catenin Signaling and Promotes Colorectal Cancer Progression *via* Interacting With DDX5. J Hematol Oncol (2018) 11(1):113. doi: 10.1186/s13045-018-0656-7 30185232 PMC6125951

[38] Zhang Y Sun J Qi Y Wang Y Ding Y Wang K. Long non-Coding RNA TPT1-AS1 Promotes Angiogenesis and Metastasis of Colorectal Cancer Through TPT1-AS1/NF90/VEGFA Signaling Pathway. Aging (2020) 12(7):6191–205. doi: 10.18632/aging.103016 PMC7185097 32248186

[39] Wang M Han D Yuan Z Hu H Zhao Z Yang R. Long non-Coding RNA H19 Confers 5-Fu Resistance in Colorectal Cancer by Promoting SIRT1-Mediated Autophagy. Cell Death Dis (2018) 9(12):1149. doi: 10.1038/s41419-018-1187-4 30451820 PMC6242979

[40] Zhang Z Yang W Li N Chen X Ma F Yang J. LncRNA MCF2L-AS1 Aggravates Proliferation, Invasion and Glycolysis of Colorectal Cancer Cells *via* the Crosstalk With miR-874-3p/FOXM1 Signaling Axis. Carcinogenesis (2021) 42(2):263–71. doi: 10.1093/carcin/bgaa093 32860508

[41] Huang F-K Zheng C-Y Huang L-K Lin C-Q Zhou J-F Wang J-X. Long non-Coding RNA MCF2L-AS1 Promotes the Aggressiveness of Colorectal Cancer by Sponging miR-874-3p and Thereby Up-Regulating CCNE1. J Gene Med (2021) 23(1):e3285. doi: 10.1002/jgm.3285 33037865

[42] Li C Wang P Du J Chen J Liu W Ye K. LncRNA RAD51-AS1/miR-29b/C-3p/NDRG2 Crosstalk Repressed Proliferation, Invasion and Glycolysis of Colorectal Cancer. IUBMB Life (2021) 73(1):286–98. doi: 10.1002/iub.2427 33314669

[43] Zhuang L Ding W Ding W Zhang Q Xu X Xi D. lncRNA ZNF667-AS1 (NR_036521.1) Inhibits the Progression of Colorectal Cancer *via* Regulating ANK2/JAK2 Expression. J Cell Physiol (2021) 236(3):2178–93. doi: 10.1002/jcp.30004 32853419

[44] Yang X-D Xu H-T Xu X-H Ru G Liu W Zhu J-J. Knockdown of Long Non-Coding RNA HOTAIR Inhibits Proliferation and Invasiveness and Improves Radiosensitivity in Colorectal Cancer. Oncol Rep (2016) 35(1):479–87. doi: 10.3892/or.2015.4397 26549670

[45] Liu Y Chen X Chen X Liu J Gu H Fan R. Long non-Coding RNA HOTAIR Knockdown Enhances Radiosensitivity Through Regulating microRNA-93/ATG12 Axis in Colorectal Cancer. Cell Death Dis (2020) 11(3):175. doi: 10.1038/s41419-020-2268-8 32144238 PMC7060216

[46] Liu T Wang H Yu H Bi M Yan Z Hong S. The Long Non-Coding RNA HOTTIP Is Highly Expressed in Colorectal Cancer and Enhances Cell Proliferation and Invasion. Mol Ther Nucleic Acids (2020) 19:612–8. doi: 10.1016/j.omtn.2019.12.008 PMC6965499 31945724

[47] Liu T Yu T Hu H He K. Knockdown of the Long non-Coding RNA HOTTIP Inhibits Colorectal Cancer Cell Proliferation and Migration and Induces Apoptosis by Targeting SGK1. Biomed Pharmacother (2018) 98:286–96. doi: 10.1016/j.biopha.2017.12.064 29274585

[48] Silva-Fisher JM Dang HX White NM Strand MS Krasnick BA Rozycki EB. Long non-Coding RNA RAMS11 Promotes Metastatic Colorectal Cancer Progression. Nat Commun (2020) 11(1):2156. doi: 10.1038/s41467-020-15547-8 32358485 PMC7195452

[49] Islam Khan MZ Law HKW. RAMS11 Promotes CRC Through mTOR-Dependent Inhibition of Autophagy, Suppression of Apoptosis, and Promotion of Epithelial-Mesenchymal Transition. Cancer Cell Int (2021) 21(1):321. doi: 10.1186/s12935-021-02023-6 34174900 PMC8236194

[50] Michael MZ OC SM van Holst Pellekaan NG Young GP James RJ. Reduced Accumulation of Specific microRNAs in Colorectal Neoplasia. Mol Cancer Res (2003) 1(12):882–91. 14573789

[51] Dong Q Zhou L Liu F Ao F Gong X Jiang C. Long non-Coding RNAs in the Development, Diagnosis and Prognosis of Nasopharyngeal Carcinoma. Int J Clin Exp Pathol (2017) 10(8):8098–105. PMC6965397 31966662

[52] Fan M Ma X Wang F Zhou Z Zhang J Zhou D. MicroRNA-30b-5p Functions as a Metastasis Suppressor in Colorectal Cancer by Targeting Rap1b. Cancer Lett (2020) 477:144–56. doi: 10.1016/j.canlet.2020.02.021 32112903

[53] Ma M Li L Long F Xiao H Lu M Lin C. MiR-133b Inhibits Colorectal Cancer Metastasis *via* lncRNA-Lucat1. Future Oncol (Lond Engl) (2021) 17(9):1013–23. doi: 10.2217/fon-2020-0420 33541136

[54] Lv L Li Q Chen S Zhang X Tao X Tang X. miR-133b Suppresses Colorectal Cancer Cell Stemness and Chemoresistance by Targeting Methyltransferase DOT1L. Exp Cell Res (2019) 385(1):111597. doi: 10.1016/j.yexcr.2019.111597 31525340

[55] Chen J Chen S Zhuo L Zhu Y Zheng H. Regulation of Cancer Stem Cell Properties, Angiogenesis, and Vasculogenic Mimicry by miR-450a-5p/SOX2 Axis in Colorectal Cancer. Cell Death Dis (2020) 11(3):173. doi: 10.1038/s41419-020-2361-z 32144236 PMC7060320

[56] Zeng Z Li Y Pan Y Lan X Song F Sun J. Cancer-Derived Exosomal miR-25-3p Promotes Pre-Metastatic Niche Formation by Inducing Vascular Permeability and Angiogenesis. Nat Commun (2018) 9(1):5395. doi: 10.1038/s41467-018-07810-w 30568162 PMC6300604

[57] Qiao P-F Yao L Zeng Z-L. Catalpol-mediated microRNA-34a Suppresses Autophagy and Malignancy by Regulating SIRT1 in Colorectal Cancer. Oncol Rep (2020) 43(4):1053–66. doi: 10.3892/or.2020.7494 PMC7057773 32323786

[58] Liu H Liu Y Sun P Leng K Xu Y Mei L. Colorectal Cancer-Derived Exosomal miR-106b-3p Promotes Metastasis by Down-Regulating DLC-1 Expression. Clin Sci (Lond Engl 1979) (2020) 134(4):419–34. doi: 10.1042/CS20191087 32065214

[59] Sun L Fang Y Wang X Han Y Du F Li C. miR-302a Inhibits Metastasis and Cetuximab Resistance in Colorectal Cancer by Targeting NFIB and CD44. Theranostics (2019) 9(26):8409–25. doi: 10.7150/thno.36605 PMC6857048 31754405

[60] Xu W Chen B Ke D Chen X. MicroRNA-138-5p Targets the NFIB-Snail1 Axis to Inhibit Colorectal Cancer Cell Migration and Chemoresistance. Cancer Cell Int (2020) 20:475. doi: 10.1186/s12935-020-01573-5 33013202 PMC7528477

[61] Bleau A-M Redrado M Nistal-Villan E Villalba M Exposito F Redin E. miR-146a Targets C-Met and Abolishes Colorectal Cancer Liver Metastasis. Cancer Lett (2018) 414:257–67. doi: 10.1016/j.canlet.2017.11.008 29133238

[62] Hwang W-L Jiang J-K Yang S-H Huang T-S Lan H-Y Teng H-W. MicroRNA-146a Directs the Symmetric Division of Snail-Dominant Colorectal Cancer Stem Cells. Nat Cell Biol (2014) 16(3):268–80. doi: 10.1038/ncb2910 24561623

[63] Lin X Wang S Sun M Zhang C Wei C Yang C. miR-195-5p/NOTCH2-Mediated EMT Modulates IL-4 Secretion in Colorectal Cancer to Affect M2-Like TAM Polarization. J Hematol Oncol (2019) 12(1):20. doi: 10.1186/s13045-019-0708-7 30808369 PMC6390326

[64] Jin Y Wang M Hu H Huang Q Chen Y Wang G. Overcoming Stemness and Chemoresistance in Colorectal Cancer Through miR-195-5p-Modulated Inhibition of Notch Signaling. Int J Biol Macromol (2018) 117:445–53. doi: 10.1016/j.ijbiomac.2018.05.151 29852230

[65] Xin H Wang C Chi Y Liu Z. MicroRNA-196b-5p Promotes Malignant Progression of Colorectal Cancer by Targeting ING5. Cancer Cell Int (2020) 20:119. doi: 10.1186/s12935-020-01200-3 32308564 PMC7149860

[66] Zhou Z Wu L Liu Z Zhang X Han S Zhao N. MicroRNA-214-3p Targets the PLAGL2-MYH9 Axis to Suppress Tumor Proliferation and Metastasis in Human Colorectal Cancer. Aging (Albany NY) (2020) 12(10):9633–57. doi: 10.18632/aging.103233 PMC7288958 32413870

[67] Li J Peng W Yang P Chen R Gu Q Qian W. MicroRNA-1224-5p Inhibits Metastasis and Epithelial-Mesenchymal Transition in Colorectal Cancer by Targeting SP1-Mediated NF-kappaB Signaling Pathways. Front Oncol (2020) 10:294. doi: 10.3389/fonc.2020.00294 32231999 PMC7083241

[68] Li SS Zhu HJ Li JY Tian LM Lv DM. MiRNA-875-3p Alleviates the Progression of Colorectal Cancer *via* Negatively Regulating PLK1 Level. Eur Rev Med Pharmacol Sci (2020) 24(3):1126–33. doi: 10.26355/eurrev_202002_20163 32096168

[69] Shahmohamadnejad S Nouri Ghonbalani Z Tahbazlahafi B Panahi G Meshkani R Emami Razavi A. Aberrant Methylation of miR-124 Upregulates DNMT3B in Colorectal Cancer to Accelerate Invasion and Migration. Arch Physiol Biochem (2020) 128:1–7. doi: 10.1080/13813455.2020.1779311 32552060

[70] Roshani Asl E Rasmi Y Baradaran B. MicroRNA-124-3p Suppresses PD-L1 Expression and Inhibits Tumorigenesis of Colorectal Cancer Cells *via* Modulating STAT3 Signaling. J Cell Physiol (2021) 236(10):7071–87. doi: 10.1002/jcp.30378 33821473

[71] Rupaimoole R Slack FJ. MicroRNA Therapeutics: Towards a New Era for the Management of Cancer and Other Diseases. Nat Rev Drug Discov (2017) 16(3):203–22. doi: 10.1038/nrd.2016.246 28209991

[72] Deng S Wang H Fan H Zhang L Hu J Tang Q. Over-Expressed miRNA-200b Ameliorates Ulcerative Colitis-Related Colorectal Cancer in Mice Through Orchestrating Epithelial-Mesenchymal Transition and Inflammatory Responses by Channel of AKT2. Int Immunopharmacol (2018) 61:346–54. doi: 10.1016/j.intimp.2018.06.024 29933193

[73] Normanno N Tejpar S Morgillo F De Luca A Van Cutsem E Ciardiello F. Implications for KRAS Status and EGFR-Targeted Therapies in Metastatic CRC. Nat Rev Clin Oncol (2009) 6(9):519–27. doi: 10.1038/nrclinonc.2009.111 19636327

[74] Ciardiello F Tortora G. EGFR Antagonists in Cancer Treatment. N Engl J Med (2008) 358(11):1160–74. doi: 10.1056/NEJMra0707704 18337605

[75] Ohta M Seto M Ijichi H Miyabayashi K Kudo Y Mohri D. Decreased Expression of the RAS-GTPase Activating Protein RASAL1 is Associated With Colorectal Tumor Progression. Gastroenterology (2009) 136(1):206–16. doi: 10.1053/j.gastro.2008.09.063 18992247

[76] Yang Y Weng W Peng J Hong L Yang L Toiyama Y. Fusobacterium Nucleatum Increases Proliferation of Colorectal Cancer Cells and Tumor Development in Mice by Activating Toll-Like Receptor 4 Signaling to Nuclear Factor-κb, and Up-Regulating Expression of MicroRNA-21. Gastroenterology (2017) 152(4):851–66. doi: 10.1053/j.gastro.2016.11.018 PMC5555435 27876571

[77] Civenni G Holbro T Hynes NE. Wnt1 and Wnt5a Induce Cyclin D1 Expression Through ErbB1 Transactivation in HC11 Mammary Epithelial Cells. EMBO Rep (2003) 4(2):166–71. doi: 10.1038/sj.embor.embor735 PMC1315833 12612606

[78] Du F Cao T Xie H Li T Sun L Liu H. KRAS Mutation-Responsive miR-139-5p Inhibits Colorectal Cancer Progression and is Repressed by Wnt Signaling. Theranostics (2020) 10(16):7335–50. doi: 10.7150/thno.45971 PMC7330859 32641995

[79] Liu T Zhang X Du L Wang Y Liu X Tian H. Exosome-Transmitted miR-128-3p Increase Chemosensitivity of Oxaliplatin-Resistant Colorectal Cancer. Mol Cancer (2019) 18(1):43. doi: 10.1186/s12943-019-0981-7 30890168 PMC6423768

[80] Xu D Wu Y Wang X Hu X Qin W Li Y. Identification of Functional circRNA/miRNA/mRNA Regulatory Network for Exploring Prospective Therapy Strategy of Colorectal Cancer. J Cell Biochem (2020) 121:4785–97. doi: 10.1002/jcb.29703 32115780

[81] Deng Q Wang CJ Hao R Yang QY. Circ_0001982 Accelerates the Progression of Colorectal Cancer *via* Sponging microRNA-144. Eur Rev Med Pharmacol Sci (2020) 24(4):1755–62. doi: 10.26355/eurrev_202002_20352 32141543

[82] Chen J Yang J Fei X Wang X Wang K. CircRNA ciRS-7: A Novel Oncogene in Multiple Cancers. Int J Biol Sci (2021) 17(1):379–89. doi: 10.7150/ijbs.54292 PMC7757028 33390857

[83] Weng W Wei Q Toden S Yoshida K Nagasaka T Fujiwara T. Circular RNA ciRS-7-A Promising Prognostic Biomarker and a Potential Therapeutic Target in Colorectal Cancer. Clin Cancer Res (2017) 23(14):3918–28. doi: 10.1158/1078-0432.CCR-16-2541 PMC5511556 28174233

[84] Santarpia L Lippman SM El-Naggar AK. Targeting the MAPK-RAS-RAF Signaling Pathway in Cancer Therapy. Expert Opin Ther Targets (2012) 16(1):103–19. doi: 10.1517/14728222.2011.645805 PMC3457779 22239440

[85] Qu S Yang X Li X Wang J Gao Y Shang R. Circular RNA: A New Star of Noncoding RNAs. Cancer Lett (2015) 365(2):141–8. doi: 10.1016/j.canlet.2015.06.003 26052092

[86] Chen L-Y Wang L Ren Y-X Pang Z Liu Y Sun X-D. The Circular RNA Circ-ERBIN Promotes Growth and Metastasis of Colorectal Cancer by miR-125a-5p and miR-138-5p/4EBP-1 Mediated Cap-Independent HIF-1α Translation. Mol Cancer (2020) 19(1):164. doi: 10.1186/s12943-020-01272-9 33225938 PMC7682012

[87] Guo L Yang G Kang Y Li S Duan R Shen L. Construction and Analysis of a ceRNA Network Reveals Potential Prognostic Markers in Colorectal Cancer. Front Genet (2020) 11:418. doi: 10.3389/fgene.2020.00418 32457800 PMC7228005

[88] Wu M Kong C Cai M Huang W Chen Y Wang B. Hsa_circRNA_002144 Promotes Growth and Metastasis of Colorectal Cancer Through Regulating miR-615-5p/LARP1/mTOR Pathway. Carcinogenesis (2020) 42:601–10. doi: 10.1093/carcin/bgaa140 PMC8086769 33347535

[89] Pan B Qin J Liu X He B Wang X Pan Y. Identification of Serum Exosomal Hsa-Circ-0004771 as a Novel Diagnostic Biomarker of Colorectal Cancer. Front Genet (2019) 10:1096. doi: 10.3389/fgene.2019.01096 31737058 PMC6838203

[90] Deng Z Li X Wang H Geng Y Cai Y Tang Y. Dysregulation of CircRNA_0001946 Contributes to the Proliferation and Metastasis of Colorectal Cancer Cells by Targeting MicroRNA-135a-5p. Front Genet (2020) 11:357. doi: 10.3389/fgene.2020.00357 32508871 PMC7232565

[91] Wang X Zhang H Yang H Bai M Ning T Deng T. Exosome-Delivered circRNA Promotes Glycolysis to Induce Chemoresistance Through the miR-122-PKM2 Axis in Colorectal Cancer. Mol Oncol (2020) 14(3):539–55. doi: 10.1002/1878-0261.12629 PMC7053238 31901148

[92] Fang G Ye BL Hu BR Ruan XJ Shi YX. CircRNA_100290 Promotes Colorectal Cancer Progression Through miR-516b-Induced Downregulation of FZD4 Expression and Wnt/beta-Catenin Signaling. Biochem Biophys Res Commun (2018) 504(1):184–9. doi: 10.1016/j.bbrc.2018.08.152 30173892

[93] Zhang L Dong X Yan B Yu W Shan L. CircAGFG1 Drives Metastasis and Stemness in Colorectal Cancer by Modulating YY1/CTNNB1. Cell Death Dis (2020) 11(7):542. doi: 10.1038/s41419-020-2707-6 32681092 PMC7367849

[94] Bian L Zhi X Ma L Zhang J Chen P Sun S. Hsa_circRNA_103809 Regulated the Cell Proliferation and Migration in Colorectal Cancer *via* miR-532-3p / FOXO4 Axis. Biochem Biophys Res Commun (2018) 505(2):346–52. doi: 10.1016/j.bbrc.2018.09.073 30249393

[95] Li C Zhou H. Circular RNA Hsa_circRNA_102209 Promotes the Growth and Metastasis of Colorectal Cancer Through miR-761-Mediated Ras and Rab Interactor 1 Signaling. Cancer Med (2020) 9(18):6710–25. doi: 10.1002/cam4.3332 PMC7520327 32706154

[96] Li R Wu B Xia J Ye L Yang X. Circular RNA Hsa_circRNA_102958 Promotes Tumorigenesis of Colorectal Cancer *via* miR-585/CDC25B Axis. Cancer Manag Res (2019) 11:6887–93. doi: 10.2147/CMAR.S212180 PMC6662515 31413634

[97] Jian X He H Zhu J Zhang Q Zheng Z Liang X. Hsa_circ_001680 Affects the Proliferation and Migration of CRC and Mediates its Chemoresistance by Regulating BMI1 Through miR-340. Mol Cancer (2020) 19(1):20. doi: 10.1186/s12943-020-1134-8 32005118 PMC6993513

[98] Wang X Ren Y Ma S Wang S. Circular RNA 0060745, a Novel circRNA, Promotes Colorectal Cancer Cell Proliferation and Metastasis Through miR-4736 Sponging. Onco Targets Ther (2020) 13:1941–51. doi: 10.2147/OTT.S240642 PMC7119088 32273712

[99] Xu H Liu Y Cheng P Wang C Liu Y Zhou W. CircRNA_0000392 Promotes Colorectal Cancer Progression Through the miR-193a-5p/PIK3R3/AKT Axis. J Exp Clin Cancer Res (2020) 39(1):283. doi: 10.1186/s13046-020-01799-1 33317596 PMC7735421

[100] Li X Wang J Zhang C Lin C Zhang J Zhang W. Circular RNA Circitga7 Inhibits Colorectal Cancer Growth and Metastasis by Modulating the Ras Pathway and Upregulating Transcription of its Host Gene ITGA7. J Pathol (2018) 246(2):166–79. doi: 10.1002/path.5125 29943828

[101] Feng J Li Z Li L Xie H Lu Q He X. Hypoxia-induced Circccdc66 Promotes the Tumorigenesis of Colorectal Cancer *via* the Mir-3140/Autophagy Pathway. Int J Mol Med (2020) 46(6):1973–82. doi: 10.3892/ijmm.2020.4747 PMC7595663 33125087

[102] Yang H Zhang H Yang Y Wang X Deng T Liu R. Hypoxia Induced Exosomal circRNA Promotes Metastasis of Colorectal Cancer *via* Targeting GEF-H1/RhoA Axis. Theranostics (2020) 10(18):8211–26. doi: 10.7150/thno.44419 PMC7381736 32724467

[103] Ma Z Han C Xia W Wang S Li X Fang P. Circ5615 Functions as a ceRNA to Promote Colorectal Cancer Progression by Upregulating TNKS. Cell Death Dis (2020) 11(5):356. doi: 10.1038/s41419-020-2514-0 32393760 PMC7214456

[104] Lu H Yao B Wen X Jia B. FBXW7 Circular RNA Regulates Proliferation, Migration and Invasion of Colorectal Carcinoma Through NEK2, mTOR, and PTEN Signaling Pathways *In Vitro* and *In Vivo* . BMC Cancer (2019) 19(1):918. doi: 10.1186/s12885-019-6028-z 31519156 PMC6744671

[105] Shang A Gu C Wang W Wang X Sun J Zeng B. Exosomal circPACRGL Promotes Progression of Colorectal Cancer *via* the miR-142-3p/miR-506-3p- TGF-Beta1 Axis. Mol Cancer (2020) 19(1):117. doi: 10.1186/s12943-020-01235-0 32713345 PMC7384220

[106] Zeng W Liu Y Li WT Li Y Zhu JF. CircFNDC3B Sequestrates miR-937-5p to Derepress TIMP3 and Inhibit Colorectal Cancer Progression. Mol Oncol (2020) 14(11):2960–84. doi: 10.1002/1878-0261.12796 PMC7607164 32896063

[107] Chen C Huang Z Mo X Song Y Li X Li X. The Circular RNA 001971/miR-29c-3p Axis Modulates Colorectal Cancer Growth, Metastasis, and Angiogenesis Through VEGFA. J Exp Clin Cancer Res (2020) 39(1):91. doi: 10.1186/s13046-020-01594-y 32430042 PMC7236474

[108] Zhang X Zhao Y Kong P Han M Li B. Expression of Circznf609 is Down-Regulated in Colorectal Cancer Tissue and Promotes Apoptosis in Colorectal Cancer Cells by Upregulating P53. Med Sci Monit (2019) 25:5977–85. doi: 10.12659/MSM.915926 PMC6703086 31401644

[109] Yuan Y Liu W Zhang Y Zhang Y Sun S. CircRNA Circ_0026344 as a Prognostic Biomarker Suppresses Colorectal Cancer Progression *via* microRNA-21 and microRNA-31. Biochem Biophys Res Commun (2018) 503(2):870–5. doi: 10.1016/j.bbrc.2018.06.089 29928882

[110] Du J Xu J Chen J Liu W Wang P Ye K. Circrae1 Promotes Colorectal Cancer Cell Migration and Invasion by Modulating miR-338-3p/TYRO3 Axis. Cancer Cell Int (2020) 20:430. doi: 10.1186/s12935-020-01519-x 32908453 PMC7470687

[111] Hanahan D Weinberg RA. Hallmarks of Cancer: The Next Generation. Cell (2011) 144(5):646–74. doi: 10.1016/j.cell.2011.02.013 21376230

[112] Li G Zhou L-N Yang H He X Duan Y Wu F. Ninjurin 2 Overexpression Promotes Human Colorectal Cancer Cell Growth and. Aging (2019) 11(19):8526–41. doi: 10.18632/aging.102336 PMC6814613 31597121

[113] Pistritto G Trisciuoglio D Ceci C Garufi A D'Orazi G. Apoptosis as Anticancer Mechanism: Function and Dysfunction of its Modulators and Targeted Therapeutic Strategies. Aging (2016) 8(4):603–19. doi: 10.18632/aging.100934 PMC4925817 27019364

[114] Krishnamurthy N Kurzrock R. Targeting the Wnt/beta-Catenin Pathway in Cancer: Update on Effectors and Inhibitors. Cancer Treat Rev (2018) 62:50–60. doi: 10.1016/j.ctrv.2017.11.002 29169144 PMC5745276

[115] Bugter JM Fenderico N Maurice MM. Mutations and Mechanisms of WNT Pathway Tumour Suppressors in Cancer. Nat Rev Cancer (2021) 21(1):5–21. doi: 10.1038/s41568-020-00307-z 33097916

[116] Yu J Han Z Sun Z Wang Y Zheng M Song C. LncRNA SLCO4A1-AS1 Facilitates Growth and Metastasis of Colorectal Cancer Through β-Catenin-Dependent Wnt Pathway. J Exp Clin Cancer Res (2018) 37(1):222. doi: 10.1186/s13046-018-0896-y 30201010 PMC6131861

[117] Wang Z Jin J. LncRNA SLCO4A1-AS1 Promotes Colorectal Cancer Cell Proliferation by Enhancing Autophagy *via* miR-508-3p/PARD3 Axis. Aging (2019) 11(14):4876–89. doi: 10.18632/aging.102081 PMC6682525 31308265

[118] Wang J Zhang Y Song H Yin H Jiang T Xu Y. The Circular RNA circSPARC Enhances the Migration and Proliferation of Colorectal Cancer by Regulating the JAK/STAT Pathway. Mol Cancer (2021) 20(1):81. doi: 10.1186/s12943-021-01375-x 34074294 PMC8167978

[119] Taherdangkoo K Kazemi Nezhad SR Hajjari MR Tahmasebi Birgani M. miR-485-3p Suppresses Colorectal Cancer *via* Targeting TPX2. Bratisl Lek Listy (2020) 121(4):302–7. doi: 10.4149/BLL_2020_048 32356447

[120] Martin TD Patel RS Cook DR Choi MY Patil A Liang AC. The Adaptive Immune System is a Major Driver of Selection for Tumor Suppressor Gene Inactivation. Science (2021) 373(6561):1327–35. doi: 10.1126/science.abg5784 PMC13397623 34529489

[121] Catalano V Turdo A Di Franco S Dieli F Todaro M Stassi G. Tumor and its Microenvironment: A Synergistic Interplay. Semin Cancer Biol (2013) 23(6 Pt B):522–32. doi: 10.1016/j.semcancer.2013.08.007 24012661

[122] Vu T Datta PK. Regulation of EMT in Colorectal Cancer: A Culprit in Metastasis. Cancers (2017) 9(12). doi: 10.3390/cancers9120171 PMC5742819 29258163

[123] Klein CA. Cancer Progression and the Invisible Phase of Metastatic Colonization. Nat Rev Cancer (2020) 20(11):681–94. doi: 10.1038/s41568-020-00300-6 33024261

[124] Fares J Fares MY Khachfe HH Salhab HA Fares Y. Molecular Principles of Metastasis: A Hallmark of Cancer Revisited. Signal Transduct Target Ther (2020) 5(1):28. doi: 10.1038/s41392-020-0134-x 32296047 PMC7067809

[125] Ji Q Cai G Liu X Zhang Y Wang Y Zhou L. MALAT1 Regulates the Transcriptional and Translational Levels of Proto-Oncogene RUNX2 in Colorectal Cancer Metastasis. Cell Death Dis (2019) 10(6):378. doi: 10.1038/s41419-019-1598-x 31097689 PMC6522477

[126] Zhou W Ye XL Xu J Cao MG Fang ZY Li LY. The lncRNA H19 Mediates Breast Cancer Cell Plasticity During EMT and MET Plasticity by Differentially Sponging miR-200b/C and Let-7b. Sci Signal (2017) 10(483). doi: 10.1126/scisignal.aak9557 28611183

[127] Liu SJ Dang HX Lim DA Feng FY Maher CA. Long Noncoding RNAs in Cancer Metastasis. Nat Rev Cancer (2021) 21(7):446–60. doi: 10.1038/s41568-021-00353-1 PMC8288800 33953369

[128] Pan M Chen Q Lu Y Wei F Chen C Tang G. MiR-106b-5p Regulates the Migration and Invasion of Colorectal Cancer Cells by Targeting FAT4. Biosci Rep (2020) 40(11). doi: 10.1042/BSR20200098 PMC7607192 33063118

[129] Shang A Wang X Gu C Liu W Sun J Zeng B. Exosomal miR-183-5p Promotes Angiogenesis in Colorectal Cancer by Regulation of FOXO1. Aging (Albany NY) (2020) 12(9):8352–71. doi: 10.18632/aging.103145 PMC7244076 32364530

[130] Herrera-Solorio AM Peralta-Arrieta I Armas Lopez L Hernandez-Cigala N Mendoza Milla C Ortiz Quintero B. LncRNA SOX2-OT Regulates AKT/ERK and SOX2/GLI-1 Expression, Hinders Therapy, and Worsens Clinical Prognosis in Malignant Lung Diseases. Mol Oncol (2021) 15(4):1110–29. doi: 10.1002/1878-0261.12875 PMC8024737 33433063

[131] Tetreault M-P Yang Y Katz JP. Krüppel-Like Factors in Cancer. Nat Rev Cancer (2013) 13(10):701–13. doi: 10.1038/nrc3582 24060862

[132] Bhattacharya R Senbanerjee S Lin Z Mir S Hamik A Wang P. Inhibition of Vascular Permeability Factor/Vascular Endothelial Growth Factor-Mediated Angiogenesis by the Kruppel-Like Factor KLF2. J Biol Chem (2005) 280(32):28848–51. doi: 10.1074/jbc.C500200200 15980434

[133] Zhang Y Li C Liu X Wang Y Zhao R Yang Y. Circhipk3 Promotes Oxaliplatin-Resistance in Colorectal Cancer Through Autophagy by Sponging miR-637. EBioMedicine (2019) 48:277–88. doi: 10.1016/j.ebiom.2019.09.051 PMC6838436 31631038

[134] Glick D Barth S Macleod KF. Autophagy: Cellular and Molecular Mechanisms. J Pathol (2010) 221(1):3–12. doi: 10.1002/path.2697 20225336 PMC2990190

[135] Singh SS Vats S Chia AY Tan TZ Deng S Ong MS. Dual Role of Autophagy in Hallmarks of Cancer. Oncogene (2018) 37(9):1142–58. doi: 10.1038/s41388-017-0046-6 29255248

[136] Chen L He M Zhang M Sun Q Zeng S Zhao H. The Role of Non-Coding RNAs in Colorectal Cancer, With a Focus on Its Autophagy. Pharmacol Ther (2021) 226:107868. doi: 10.1016/j.pharmthera.2021.107868 33901505

[137] Pentimalli F. Autophagy in Disease: Hunger for Translation. Cell Death Dis (2019) 10(3):247. doi: 10.1038/s41419-019-1419-2 30867407 PMC6416282

[138] Sun W Li J Zhou L Han J Liu R Zhang H. The C-Myc/miR-27b-3p/ATG10 Regulatory Axis Regulates Chemoresistance in Colorectal Cancer. Theranostics (2020) 10(5):1981–96. doi: 10.7150/thno.37621 PMC7019154 32104496

[139] Liu P-F Farooqi AA Peng S-Y Yu T-J Dahms H-U Lee C-H. Regulatory Effects of Noncoding RNAs on the Interplay of Oxidative Stress and Autophagy in Cancer Malignancy and Therapy. Semin Cancer Biol (2020). doi: 10.1016/j.semcancer.2020.10.009 33127466

[140] Liu F Ai FY Zhang DC Tian L Yang ZY Liu SJ. LncRNA NEAT1 Knockdown Attenuates Autophagy to Elevate 5-FU Sensitivity in Colorectal Cancer *via* Targeting miR-34a. Cancer Med-Us (2019) 9(3):1079–91. doi: 10.1002/cam4.2746 PMC6997058 31802650

[141] Che J Wang W Huang Y Zhang L Zhao J Zhang P. miR-20a Inhibits Hypoxia-Induced Autophagy by Targeting ATG5/FIP200 in Colorectal Cancer. Mol Carcinog (2019) 58(7):1234–47. doi: 10.1002/mc.23006 30883936

[142] Cotton PB Durkalski VL Pineau BC Palesch YY Mauldin PD Hoffman B. Computed Tomographic Colonography (Virtual Colonoscopy): A Multicenter Comparison With Standard Colonoscopy for Detection of Colorectal Neoplasia. JAMA (2004) 291(14):1713–9. doi: 10.1001/jama.291.14.1713 15082698

[143] Moss S Mathews C Day TJ Smith S Seaman HE Snowball J. Increased Uptake and Improved Outcomes of Bowel Cancer Screening With a Faecal Immunochemical Test: Results From a Pilot Study Within the National Screening Programme in England. Gut (2017) 66(9):1631–44. doi: 10.1136/gutjnl-2015-310691 27267903

[144] Zhu YX Xu AD Li JM Fu JH Wang GH Yang YL. Fecal miR-29a and miR-224 as the Noninvasive Biomarkers for Colorectal Cancer. Cancer Biomark (2016) 16(2):259–64. doi: 10.3233/Cbm-150563 PMC13016455 26756616

[145] Ling H Pickard K Ivan C Isella C Ikuo M Mitter R. The Clinical and Biological Significance of MIR-224 Expression in Colorectal Cancer Metastasis. Gut (2016) 65(6):977–89. doi: 10.1136/gutjnl-2015-309372 PMC4581915 25804630

[146] Li T Lai Q Wang S Cai J Xiao Z Deng D. MicroRNA-224 Sustains Wnt/beta-Catenin Signaling and Promotes Aggressive Phenotype of Colorectal Cancer. J Exp Clin Cancer Res (2016) 35:21. doi: 10.1186/s13046-016-0287-1 26822534 PMC4731927

[147] Hur K Toiyama Y Okugawa Y Ide S Imaoka H Boland CR. Circulating microRNA-203 Predicts Prognosis and Metastasis in Human Colorectal Cancer. Gut (2017) 66(4):654–65. doi: 10.1136/gutjnl-2014-308737 PMC4919275 26701878

[148] Pidikova P Reis R Herichova I. miRNA Clusters With Down-Regulated Expression in Human Colorectal Cancer and Their Regulation. Int J Mol Sci (2020) 21(13). doi: 10.3390/ijms21134633 PMC7369991 32610706

[149] Micallef I Baron B. The Mechanistic Roles of ncRNAs in Promoting and Supporting Chemoresistance of Colorectal Cancer. Noncoding RNA (2021) 7(2). doi: 10.3390/ncrna7020024 PMC8103280 33807355

[150] Li P Zhang X Wang H Wang L Liu T Du L. MALAT1 Is Associated With Poor Response to Oxaliplatin-Based Chemotherapy in Colorectal Cancer Patients and Promotes Chemoresistance Through EZH2. Mol Cancer Ther (2017) 16(4):739–51. doi: 10.1158/1535-7163.MCT-16-0591 28069878

[151] Fanale D Castiglia M Bazan V Russo A. Involvement of Non-Coding RNAs in Chemo- and Radioresistance of Colorectal Cancer. Adv Exp Med Biol (2016) 937:207–28. doi: 10.1007/978-3-319-42059-2_11 27573902

[152] Wei L Wang X Lv L Zheng Y Zhang N Yang M. The Emerging Role of Noncoding RNAs in Colorectal Cancer Chemoresistance. Cell Oncol (Dordr) (2019) 42(6):757–68. doi: 10.1007/s13402-019-00466-8 PMC12994294 31359293

[153] Barisciano G Colangelo T Rosato V Muccillo L Taddei ML Ippolito L. miR-27a is a Master Regulator of Metabolic Reprogramming and Chemoresistance in Colorectal Cancer. Br J Cancer (2020) 122(9):1354–66. doi: 10.1038/s41416-020-0773-2 PMC7188668 32132656

[154] Yang H Li X Meng Q Sun H Wu S Hu W. CircPTK2 (Hsa_Circ_0005273) as a Novel Therapeutic Target for Metastatic Colorectal Cancer. Mol Cancer (2020) 19(1):13. doi: 10.1186/s12943-020-1139-3 31973707 PMC6977296

[155] Xu J Zhang G Luo X Wang D Zhou W Zhang Y. Co-Delivery of 5-Fluorouracil and miRNA-34a Mimics by Host-Guest Self-Assembly Nanocarriers for Efficacious Targeted Therapy in Colorectal Cancer Patient-Derived Tumor Xenografts. Theranostics (2021) 11(5):2475–89. doi: 10.7150/thno.52076 PMC7797688 33500737

[156] Liu Y Zhang M Liang L Li J Chen YX. Over-Expression of lncRNA DANCR is Associated With Advanced Tumor Progression and Poor Prognosis in Patients With Colorectal Cancer. Int J Clin Exp Pathol (2015) 8(9):11480–4. PMC4637695 26617879

[157] Liang ZX Liu HS Wang FW Xiong L Zhou C Hu T. LncRNA RPPH1 Promotes Colorectal Cancer Metastasis by Interacting With TUBB3 and by Promoting Exosomes-Mediated Macrophage M2 Polarization. Cell Death Dis (2019) 10(11):829. doi: 10.1038/s41419-019-2077-0 31685807 PMC6828701

[158] Zhou C Liu HS Wang FW Hu T Liang ZX Lan N. Circcamsap1 Promotes Tumor Growth in Colorectal Cancer *via* the miR-328-5p/E2F1 Axis. Mol Ther (2020) 28(3):914–28. doi: 10.1016/j.ymthe.2019.12.008 PMC7054739 31951832

[159] Zhang Z Zhou C Chang Y Zhang Z Hu Y Zhang F. Long non-Coding RNA CASC11 Interacts With hnRNP-K and Activates the WNT/beta-Catenin Pathway to Promote Growth and Metastasis in Colorectal Cancer. Cancer Lett (2016) 376(1):62–73. doi: 10.1016/j.canlet.2016.03.022 27012187

[160] Mai D Ding P Tan L Zhang J Pan Z Bai R. PIWI-Interacting RNA-54265 is Oncogenic and a Potential Therapeutic Target in Colorectal Adenocarcinoma. Theranostics (2018) 8(19):5213–30. doi: 10.7150/thno.28001 PMC6276099 30555542

[161] Fang X Yang D Luo H Wu S Dong W Xiao J. SNORD126 Promotes HCC and CRC Cell Growth by Activating the PI3K-AKT Pathway Through FGFR2. J Mol Cell Biol (2017) 9(3):243–55. doi: 10.1093/jmcb/mjw048 27913571

[162] Thompson DM Parker R. Stressing Out Over tRNA Cleavage. Cell (2009) 138(2):215–9. doi: 10.1016/j.cell.2009.07.001 19632169

[163] Goodarzi H Liu X Nguyen HCB Zhang S Fish L Tavazoie SF. Endogenous tRNA-Derived Fragments Suppress Breast Cancer Progression *via* YBX1 Displacement. Cell (2015) 161(4):790–802. doi: 10.1016/j.cell.2015.02.053 25957686 PMC4457382

[164] Jürchott K Kuban R-J Krech T Blüthgen N Stein U Walther W. Identification of Y-Box Binding Protein 1 as a Core Regulator of MEK/ERK Pathway-Dependent Gene Signatures in Colorectal Cancer Cells. PloS Genet (2010) 6(12):e1001231. doi: 10.1371/journal.pgen.1001231 21170361 PMC2996331

[165] Luan N Chen Y Li Q Mu Y Zhou Q Ye X. TRF-20-M0NK5Y93 Suppresses the Metastasis of Colon Cancer Cells by Impairing the Epithelial-to-Mesenchymal Transition Through Targeting Claudin-1. Am J Trans Res (2021) 13(1):124–42. PMC7847510 33527013

[166] Huang B Yang H Cheng X Wang D Fu S Shen W. tRF/miR-1280 Suppresses Stem Cell-Like Cells and Metastasis in Colorectal Cancer. Cancer Res (2017) 77(12):3194–206. doi: 10.1158/0008-5472.CAN-16-3146 28446464

[167] Huang J-Z Chen M Chen D Gao X-C Zhu S Huang H. A Peptide Encoded by a Putative lncRNA HOXB-AS3 Suppresses Colon Cancer Growth. Mol Cell (2017) 68(1):171–84. doi: 10.1016/j.molcel.2017.09.015 28985503

[168] Wang L Zhou J Zhang C Chen R Sun Q Yang P. A Novel Tumour Suppressor Protein Encoded by Circmapk14 Inhibits Progression and Metastasis of Colorectal Cancer by Competitively Binding to MKK6. Clin Trans Med (2021) 11(10):e613. doi: 10.1002/ctm2.613 PMC8516360 34709743

[169] Dong J Tai JW Lu L-F. miRNA-Microbiota Interaction in Gut Homeostasis and Colorectal Cancer. Trends Cancer (2019) 5(11):666–9. doi: 10.1016/j.trecan.2019.08.003 PMC8480531 31735285

[170] Tilg H Adolph TE Gerner RR Moschen AR. The Intestinal Microbiota in Colorectal Cancer. Cancer Cell (2018) 33(6):954–64. doi: 10.1016/j.ccell.2018.03.004 29657127

